# Development and evaluation of nomograms and risk stratification systems to predict the overall survival and cancer-specific survival of patients with hepatocellular carcinoma

**DOI:** 10.1007/s10238-024-01296-1

**Published:** 2024-02-28

**Authors:** Xichun Kang, Xiling Liu, Yaoqi Li, Wenfang Yuan, Yi Xu, Huimin Yan

**Affiliations:** 1 Department of Epidemiology and Health Statistics, School of Public Health, Hebei Medical University, Shijiazhuang, 050017 China; 2 Clinical Research Center, The Fifth Hospital of Shijiazhuang, Shijiazhuang, 050021 China; 3 Department of the Sixth Infection, The Fifth Hospital of Shijiazhuang, Shijiazhuang, 050021 China; 4 Department of Laboratory Medicine, The Fifth Hospital of Shijiazhuang, Shijiazhuang, 050021 China

**Keywords:** Hepatocellular carcinoma, Overall survival, Cancer-specific survival, Nomogram, Risk stratification

## Abstract

Hepatocellular carcinoma (HCC) is the most common type of primary liver cancer, and patients with HCC have a poor prognosis and low survival rates. Establishing a prognostic nomogram is important for predicting the survival of patients with HCC, as it helps to improve the patient’s prognosis. This study aimed to develop and evaluate nomograms and risk stratification to predict overall survival (OS) and cancer-specific survival (CSS) in HCC patients. Data from 10,302 patients with initially diagnosed HCC were extracted from the Surveillance, Epidemiology, and End Results (SEER) database between 2010 and 2017. Patients were randomly divided into the training and validation set. Kaplan–Meier survival, LASSO regression, and Cox regression analysis were conducted to select the predictors of OS. Competing risk analysis, LASSO regression, and Cox regression analysis were conducted to select the predictors of CSS. The validation of the nomograms was performed using the concordance index (C-index), the Akaike information criterion (AIC), the Bayesian information criterion (BIC), Net Reclassification Index (NRI), Discrimination Improvement (IDI), the receiver operating characteristic (ROC) curve, calibration curves, and decision curve analyses (DCAs). The results indicated that factors including age, grade, T stage, N stage, M stage, surgery, surgery to lymph node (LN), Alpha-Fetal Protein (AFP), and tumor size were independent predictors of OS, whereas grade, T stage, surgery, AFP, tumor size, and distant lymph node metastasis were independent predictors of CSS. Based on these factors, predictive models were built and virtualized by nomograms. The C-index for predicting 1-, 3-, and 5-year OS were 0.788, 0.792, and 0.790. The C-index for predicting 1-, 3-, and 5-year CSS were 0.803, 0.808, and 0.806. AIC, BIC, NRI, and IDI suggested that nomograms had an excellent predictive performance with no significant overfitting. The calibration curves showed good consistency of OS and CSS between the actual observation and nomograms prediction, and the DCA showed great clinical usefulness of the nomograms. The risk stratification of OS and CSS was built that could perfectly classify HCC patients into three risk groups. Our study developed nomograms and a corresponding risk stratification system predicting the OS and CSS of HCC patients. These tools can assist in patient counseling and guiding treatment decision making.

**Supplementary Information:**

The online version contains supplementary material available at 10.1007/s10238-024-01296-1.

## Introduction

Primary liver cancer is the sixth most common cancer and the fourth leading cause of cancer death worldwide [1]. Its incidence is increasing and more than 1 million people are estimated to die in 2030 [2, 3]. Hepatocellular carcinoma (HCC) accounts for > 80% of primary liver cancers. Almost 85% of HCC cases are estimated to arise in sub-Saharan Africa and Eastern Asia with China accounting for over half of new cases [4, 5]. Notably, the incidence and mortality rates of HCC are also escalating in some developed regions in Europe and the USA [6]. Despite ongoing research and advances in therapeutic strategies for HCC, the outcome for patients remains poor, with reported five-year survival rates of 14.1% and > 17% in China and the USA, respectively [7]. The reasons for this outcome are manifold, including the inefficiency of screening tools, the late diagnosis of most cases, and the lack of an effective treatment [1, 2]. The study on the prognosis of HCC patients is a hotspot because more effective treatment strategies need to be based on information regarding prognostic risks. Moreover, it is necessary to integrate multiple prognostic factors into an easy-to-use predictive system to better inform oncologists and more accurately stratify patients.

The selection of prognostic factors is crucial in the prognostic warning of HCC, but the difficulty lies in the combination of various factors. The traditional staging system has a combination of prognostic factors but is not comprehensive. There are more than 18 classification systems for staging liver cancer, such as the Barcelona Clinic Liver Cancer (BCLC) and American Joint Committee on Cancer (AJCC) staging [1]. The AJCC staging is undoubtedly an important system, which was established based on the tumor dimension criterion. However, this system is deficient in key prognostic factors such as grade and AFP. The BCLC classification system is among the most widely used classifications for HCC, but it is relatively intricate and does not consider AFP as an important prognostic factor. Given these limitations, molecular-based models have been the targets of several research efforts, with some studies demonstrating the predictive capability of circRNA, miRNA, and DNA markers in determining the prognosis of HCC patients [8, 9]. However, these molecular markers have significant variations, making them less accurate and highly costly. In previous studies, some prognostic factors for HCC patients have been reported, including stage, AFP, and metastatic status [10–12]. Of note, substantial heterogeneity exists among patients with HCC in terms of demographic and clinicopathological information [1]. Consequently, the prognosis of HCC can vary considerably across different cases. Moreover, the effect of treatments can differ considerably among different patients, so treatments have been incorporated as prognostic factors to ascertain their benefits for patients and avoid overtreatment.

The Surveillance, Epidemiology, and End Results (SEER) program is a clinical database, funded by the National Cancer Institute (NCI), which was established to collect cancer incidence, prevalence, and survival data from US cancer registries. The SEER database is a multicenter patients-based database containing comprehensive clinical data on different types of malignancies, such as patient information, location of the primary lesion, tumor size, treatment, and cause of death. In this study, we used data from the SEER database to construct and evaluate nomograms of overall survival (OS) and cancer-specific survival (CSS) in patients with HCC [13].

A nomogram is a predictive tool that generates a graph based on a predictive statistical model. By integrating diverse prognostic and determinant variables to produce an individual probability of a clinical event, nomograms satisfy our need for biologically and clinically integrated models and advance our pursuit of personalized medicine [14]. The rapid computation of nomograms through user-friendly digital interfaces, along with enhanced accuracy and easily interpretable prognosis compared to conventional staging, facilitates the smooth integration of nomogram-derived prognosis into clinical decision making [15]. In this study, we aimed to incorporate important factors obtained from the SEER database to develop and validate nomograms for predicting the OS and CSS of patients with HCC. We hoped to help clinicians better identify the individual’s risk of death and make adjustments to their current treatment accurately and beneficially.

## Material and methods 

### Ethics statement 

The SEER database is a public database and has patient anonymization. Thus, our study was exempt from the ethical review or patient consent.

### Data acquisition and collection

We extracted data from the SEER database (Incidence-SEER Research Plus Data, 18 Registries, Nov 2020 Sub (2000–2018)) using the SEER*Stat program (version 8.3.9). Because AFP data were only available after 2010, we included patients with HCC diagnosed between 2010 and 2017. Patients with HCC were randomly assigned to the training and validation groups in a 7:3 ratio. The training set was used to develop nomograms and a risk stratification system, while the validation set was used to assess the performance of the nomograms.

### Patient selection criteria and prognostic variables

Inclusion criteria were listed as follows: (1) patients diagnosed with HCC ((histological type ICD-O-3 = 8170–8175) between 2010 and 2017, (2) complete clinical, therapy, staging system, and metastasis information, (3) pathological diagnosis available, (4) clear survival time and histological grade.

**Fig. 1:**
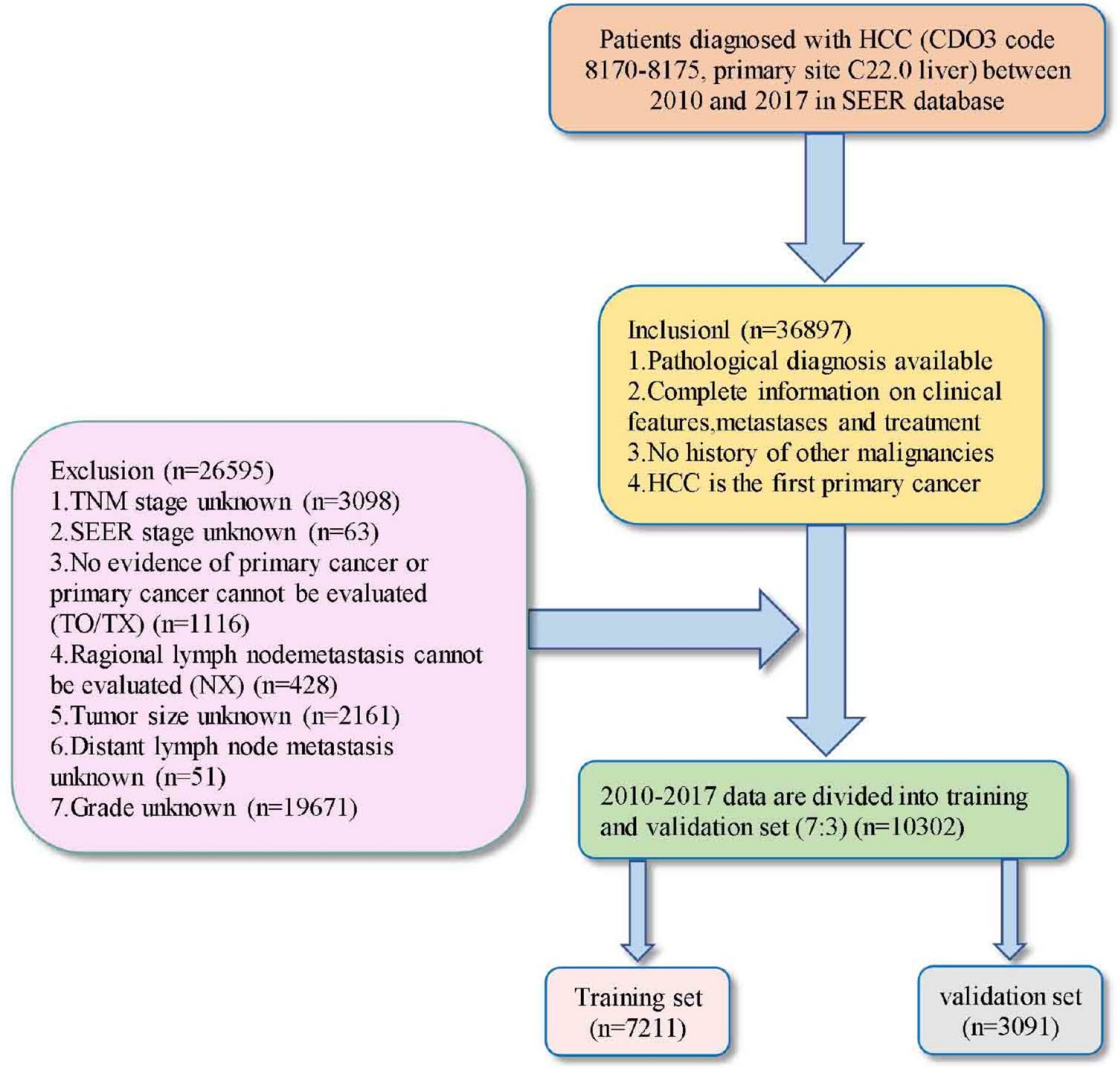
The flow diagram of the selection process for the study

Exclusion criteria were listed as follows: (1) HCC is not the first primary cancer and history of other cancer, (2) no evidence of primary cancer or primary cancer cannot be evaluated (T0/TX) and regional lymph node (LN) metastasis cannot be evaluated (NX), (3) tumor size unknown. The screening flowchart is shown in Fig. 1.

**Fig. 2:**
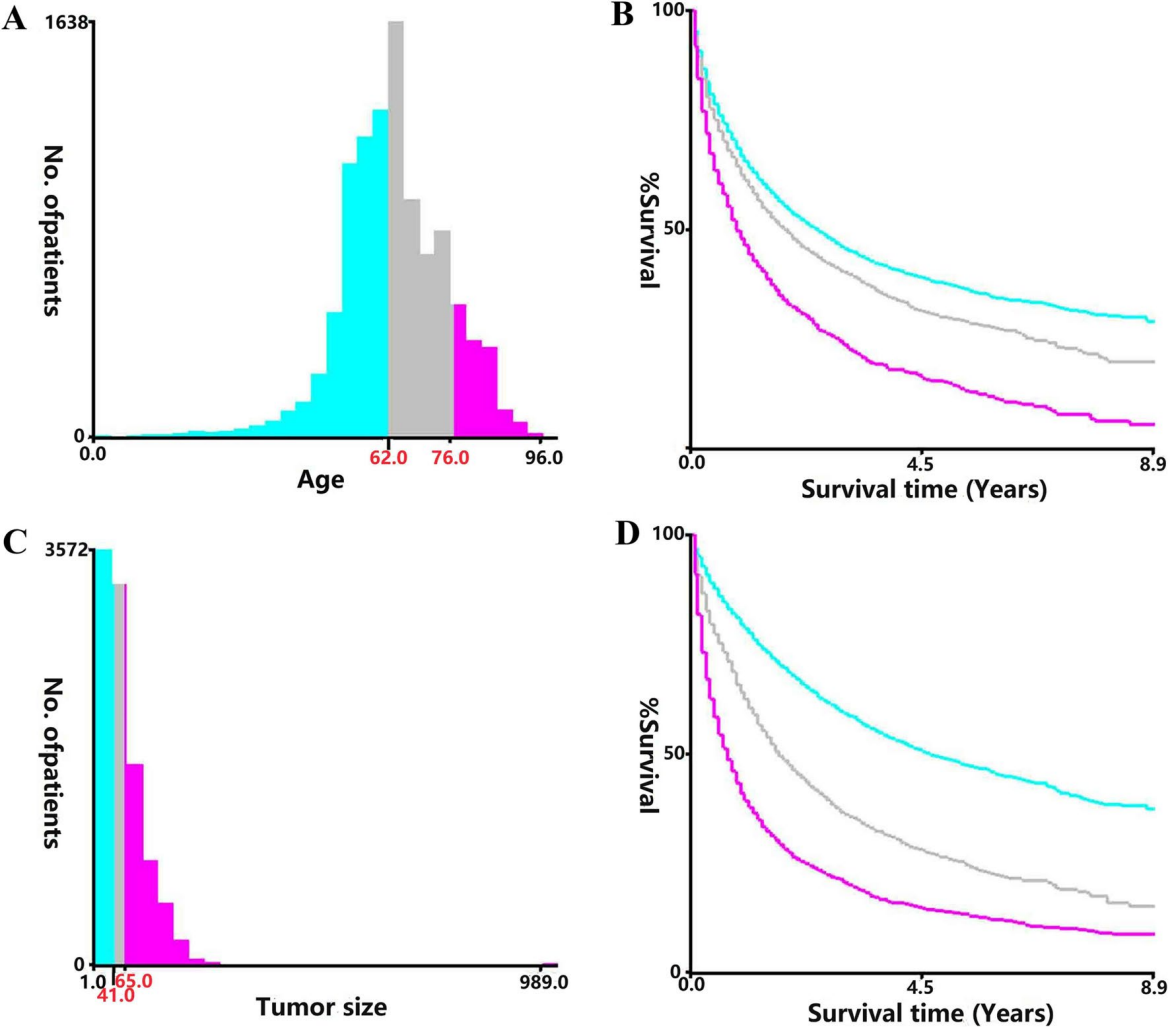
The graphs show defining the optimal cutoff values of age and tumor size via X-tile analysis. A histogram (**A**, **C**) and Kaplan–Meier survival curve (**B**, **D**) were constructed based on the identified cutoff values

The following categories were selected for our research: age, sex, race, marital status, grade, AJCC staging, SEER staging, surgery, surgery to LN, radiation, chemotherapy, AFP, tumor size, bone metastasis, brain metastasis, liver metastasis, lung metastasis, distant LN metastasis. Continuous variables such as age and tumor size are classified into three categories based on the X-tile software (Fig. 2).

### Endpoint definition

The primary endpoints of the study were OS and CSS. OS was defined as the interval between the initial diagnosis of HCC and the occurrence of death from any cause. The CSS was defined as the interval between the initial diagnosis of HCC and the occurrence of HCC-specific death. It should be noted that for patients with a survival time of less than one month, their survival time was set to zero in the SEER database. Therefore, we assigned a survival time of 0.5 to these patients.

### The development and validation of the prognostic nomograms

We screened prognostic variables for OS and CSS using Kaplan–Meier (KM) analysis and competing risk analysis, respectively. A competing risk is an event that prevents the occurrence of the primary event of interest [16]. In the competing risk model, variables with a *P *value of less than 0.2 were included in univariate Cox analysis to further filter the variables. Subsequently, to minimize the possibility of overfitting the models, LASSO regression was used. LASSO regression sets the coefficients of many irrelevant variables to zero based on a penalty parameter *λ* [17]. The results of LASSO regression were included in multivariate Cox regression analysis. Finally, we constructed nomograms for OS and CSS based on the multivariate Cox regression model.

We assessed the performance of the nomograms for OS and CSS using several indices. Concordance index (C-index), time-dependent area under the receiver operating characteristic curve (AUC), and employing receiver operating characteristic curves (ROC) were used to measure the discriminative ability of the model, with values ranging from 0 to 1. Higher values indicated the better predictive performance of the nomograms. Akaike information criterion (AIC) [18] and Bayesian information criterion (BIC) [19] were used to evaluate the model’s goodness of fit, with lower values indicating better predictive performance. Net Reclassification Index (NRI) was used to assess the model’s predictive performance [20], and Integrated Discrimination Improvement (IDI) [21] was used to compare the model’s overall improvement. The predictive accuracy of the nomograms was further examined using calibration curves. The calibration curve could be used to observe the agreement between the predicted values of nomograms and actual values. In the analysis of the calibration curve, the prediction error was estimated using the nonparametric bootstrap procedure with 1000 bootstrap replicates to minimize bias. To evaluate the usefulness and benefit of the nomograms, a decision curve analysis (DCA) was performed [22]. The OS and CSS nomograms were subjected to 1,000 bootstraps resamples for internal validation.

### Risk stratification for HCC patients

The risk score for each HCC patient was determined by the risk stratification. The cutoff value of the total score was analyzed using the X-tile software to distinguish the differences in OS and CSS of HCC patients. Based on the established prognostic nomograms, HCC patients were stratified into high-, intermediate-, and low-risk groups. The KM method was employed to estimate the survival function for different risk groups, and the log-rank test was utilized to evaluate the statistical significance of the results. Additionally, a stacked bar chart was employed to further illustrate the ontological characteristics of HCC patients within different risk stratifications. To further verify the stability and performance of the nomograms from different dimensions and investigate whether specific subgroups were prone to errors, we divided patients into different subgroups. The KM survival curves for each subgroup were generated.

### Statistical analysis

The Chi-square test was used to identify the significance of the differences among the categorical variables in the training and validation set. All variable values were presented as quantity (*n*) and percentage (%) of cases after sorting them out into baseline characteristics.

All data were analyzed using SPSS 22.0 and the R software (version 4.1.2, http://www.r-project.org/). A *P* value ≤ 0.05 (two-sided) was considered statistically significant. The R packages were used to develop and validate nomograms as follows: “survival,” “glmnet,” “rms,” “performance,” “riskRegression,” “pec,” “survIDINRI,” “nricens,” “survminer,” “ggplot2,” “cmprsk.”

## Results

### Baseline characteristics of HCC patients

A total of 10,302 HCC patients were screened out from the SEER database, which was divided into a training set (*n* = 7211) and a validation set (*n* = 3091). As shown in Table 1, there was no significant difference between the two sets of clinical characteristics, oncology features, cancer metastasis, and cancer therapy (*P* > 0.05). Regarding the composition of HCC patients in the training set, the majority of the HCC patients were age ≤ 62.00 (46.5%), male (76.6%), white (66.6%), married (78.7%), grade I–II (77.4%), AJCC staging I (43.0%), localized (61.1%), *T*1 (46.1%), N0 (93.1%), M0 (89.8%), surgery—no receive (51.8%), LN surgery—no received (92.0%), radiation—no received (90.2%), chemotherapy—no received (63.8%), bone metastasis—no occurred (97.5%), brain metastasis-no occurred (99.7%), liver metastasis-no occurred (99.3%), lung metastasis-no occurred (95.9%), distant LN metastasis- no occurred (91.7%), tumor size ≤ 41.00 mm (42.4%), and AFP positive (54.1%). These features still existed in the validation cohort and total cohort.

**Table 1:** Baseline clinicopathological characteristics of all patients and those in the training and validation set

Variable	All patients, *n* (%)	Training set, *n* (%)	Validation set, *n* (%)	χ^2^	*P* value
Number of patients	10,302 (100)	7211 (100)	3091 (100)		
Age, years median (IQR)	63 (57–71)	63 (57–71)	63 (57–71)		
Age				1.940	0.380
≤ 62	4764 (46.2)	3328 (46.2)	1436 (46.5)		
63–76	4107 (39.9)	2900 (40.2)	1207 (39.0)		
≥ 77	1431 (13.9)	983 (13.6)	448 (14.5)		
Sex				1.521	0.225
Female	2445 (23.7)	1687 (23.4)	758 (24.5)		
Male	7857 (76.3)	5524 (76.6)	2333 (75.5)		
Race				0.323	0.851
White	6843 (66.4)	4802 (66.6)	2041 (66.0)		
Black	1411 (13.7)	981 (13.6)	430 (13.9)		
Other^a^	2048 (19.9)	1428 (19.8)	620 (20.1)		
Marital status				0.005	0.958
Married	8108 (78.7)	5674 (78.7)	2434 (78.7)		
Unmarried	2194 (21.3)	1537 (21.3)	657 (21.3)		
Grade				0.004	0.959
Grade I–II	7975 (77.4)	5581 (77.4)	2394 (77.5)		
Grade III–IV	2327 (22.6)	1630 (22.6)	697 (22.5)		
AJCC,7th				0.928	0.819
I	4432 (43.0)	3104 (43.0)	1328 (43.0)		
II	2302 (22.3)	1613 (22.4)	689 (22.3)		
III	2147 (20.8)	1488 (20.6)	659 (21.3)		
IV	1421 (13.8)	1006 (14.0)	415 (13.4)		
T stage				0.129	0.988
T1	4758 (46.2)	3325 (46.1)	1433 (46.4)		
T2	2509 (24.4)	1756 (24.4)	753 (24.4)		
T3	2643 (25.7)	1853 (25.7)	790 (25.6)		
T4	392 (3.8)	277 (3.8)	115 (3.7)		
N stage				0.037	0.865
N0	9593 (93.1)	6717 (93.1)	2876 (93.0)		
N1	709 (6.9)	494 (6.9)	215 (7.0)		
M stage				1.099	0.299
M0	9270 (90.0)	6474 (89.8)	2796 (90.5)		
M1	1032 (10.0)	737 (10.2)	295 (9.5)		
SEER Stage				2.025	0.364
Localized	6339 (61.5)	4405 (61.1)	1934 (62.6)		
Regional	2854 (27.7)	2019 (28.0)	835 (27.0)		
Distant	1109 (10.8)	787 (10.9)	322 (10.4)		
Surgery				1.333	0.932
No surgery	5346 (51.9)	3734 (51.8)	1612 (52.2)		
Destruction	1100 (10.7)	766 (10.6)	334 (10.8)		
Resection	1610 (15.6)	1118 (15.5)	492 (15.9)		
Lobectomy	1121 (10.9)	793 (11.0)	328 (10.6)		
Hepatectomy	1107 (10.7)	787 (10.9)	320 (10.4)		
Transplant	18 (0.2)	13 (0.2)	5 (0.2)		
Surgery to LN^b^				0.003	0.968
Yes	819 (7.9)	574 (8.0)	245 (7.9)		
No	9483 (92.1)	6637 (92.0)	2846 (92.1)		
Radiation				0.492	0.495
Yes	1024 (9.9)	707 (9.8)	317 (10.3)		
No	9278 (90.1)	6504 (90.2)	2774 (89.7)		
Chemotherapy				0.194	0.671
Yes	3719 (36.1)	2613 (36.2)	1106 (35.8)		
No	6583 (63.9)	4598 (63.8)	1985 (64.2)		
AFP				3.474	0.176
Negative	2787 (27.1)	1949 (27.0)	838 (27.1)		
Positive	5620 (54.6)	3903 (54.1)	1717 (55.5)		
Unknown	1895 (18.4)	1359 (18.8)	536 (17.3)		
Tumor Size(mm)				1.362	0.506
v≤ 41	4356 (42.3)	3060 (42.4)	1296 (41.9)		
42–65	2311 (22.4)	1595 (22.1)	716 (23.2)		
≥ 66	3635 (35.3)	2566 (35.4)	1079 (34.9)		
Bone metastasis				0.490	0.487
Yes	250 (2.4)	180 (2.5)	70 (2.3)		
No	10,052 (97.6)	7031 (97.5)	3021 (97.7)		
Brain metastasis				0.335	0.682
Yes	28 (0.3)	21 (0.3)	7 (0.2)		
No	10,274 (99.7)	7190 (99.7)	3084 (99.8)		
Liver metastasis				1.050	0.347
Yes	66 (0.6)	50 (0.7)	16 (0.5)		
No	10,236 (99.4)	7161 (99.3)	3075 (99.5)		
Lung metastasis				0.095	0.785
Yes	416 (4.0)	294 (4.1)	122 (3.9)		
No	9886 (96.0)	6917 (95.9)	2969 (96.1)		
Distant LN metastasis				0.369	0.556
Yes	839 (8.1)	595 (8.3)	244 (7.9)		
No	9463 (91.9)	6616 (91.7)	2847 (92.1)		
Cancer-specific survival				1.765	0.413
Alive	3662 (35.5)	2584 (35.8)	1078 (34.9)		
Death due to cancer	4704 (45.7)	3294 (45.7)	1410 (45.6)		
Death due to other reasons	1936 (18.8)	1333 (18.5)	603 (19.5)		
Overall survival				0.868	0.357
Alive	3662 (35.5)	2584 (35.8)	1078 (34.9)		
Dead	6640 (64.5)	4627 (64.2)	2013 (65.1)		

^a^Other (American Indian/AK Native, Asian/Pacific Islander)

^b^Lymph node = LN

The median survival time of HCC patients in the training set was 22.00 (5.00, 90.00) months. A total of 2584 patients were alive (35.8%) and 4,627 patients were dead (64.2%). For the death cause of patients, 3294 HCC patients died of cancer (45.7%) and 1,333 HCC patients died of other reasons.

### Identification of prognostic factors of HCC patients

**Fig. 3:**
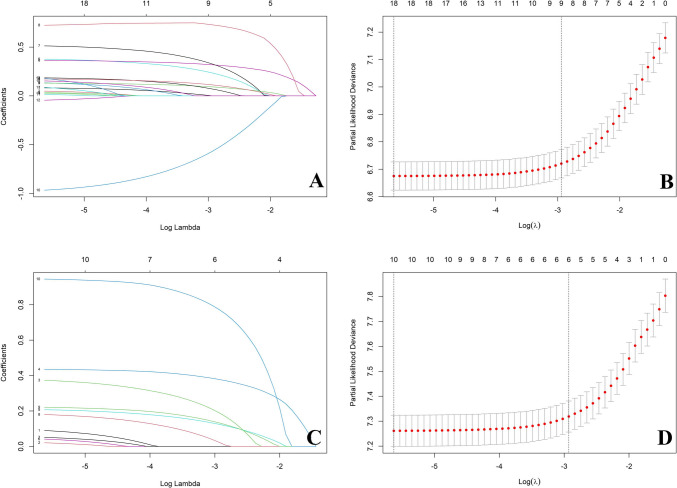
Prognostic variables of overall survival (OS) and cancer-specific survival (CSS) selection using the LASSO regression analysis. **A**–**B** LASSO regression analysis for the prognostic variables of overall survival (OS), **C**–**D** LASSO regression analysis for the prognostic variables of cancer-specific survival (CSS)

**Fig. 4:**
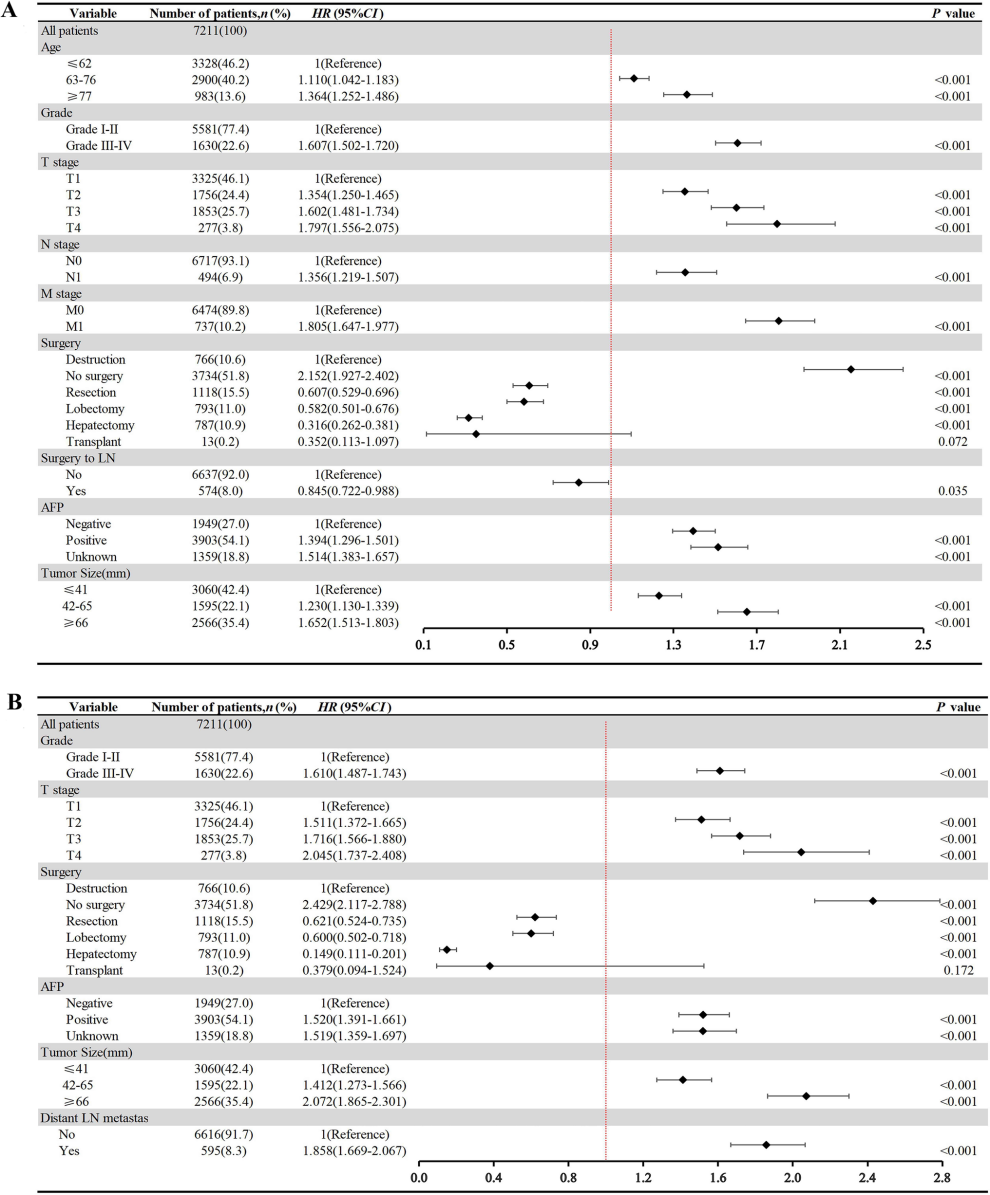
Forest plots of multivariable Cox regression result for the selection of prognostic variables of overall survival (OS) (**A**) and cancer-specific survival (CSS) (**B**)

The KM survival analysis for OS showed that 19 variables were statistical significance. The median survival time and OS probability for each subgroup were listed (Supplementary Table 1). LASSO regression and multivariate Cox regression analysis indicated that the variables, including age, grade, T stage, N stage, M stage, surgery, surgery to LN, AFP, and tumor size, have an impact on the OS of HCC patients. (Figs. 3A–B, 4A).

According to the competing risk analysis and univariate Cox analysis, sex, race, grade, T stage, surgery, radiation, chemotherapy, AFP, tumor size, and distant LN metastasis were related to CSSs (Table 2). The cumulative mortality curves were determined based on deaths from cancer and other causes in HCC patients (Supplementary Fig. 1). LASSO regression and multivariate Cox regression analysis indicated that the variables, including grade, T stage, surgery, AFP, tumor size, and distant LN metastasis as significant variables for CSS (Figs. 3C–D, 4B).

**Table 2:** Results of univariate and multivariate competing risk models and univariate Cox regression for cancer-specific survival

Variable	Number of patients, *n* (%)	Cancer-specific survival					
		Competing risk analysis				Cox regression analysis	
		Univariate		Multivariate		Univariate analysis	
		SHR (95% CI)	*P* value	SHR (95% CI)	*P* value	HR (95% CI)	*P* value
All patients	7211 (100)						
Age							
≤ 62	3328 (46.2)	1 (Reference)		1 (Reference)			
63–76	2900 (40.2)	1.203 (1.121–1.285)	< 0.001	0.964 (0.889–1.044)	0.360		
≥ 77	983 (13.6)	1.668 (1.500–1.836)	< 0.001	1.057 (0.946–1.182)	0.330		
Sex							
Female	1687 (23.4)	1 (Reference)		1 (Reference)		1 (Reference)	
Male	5524 (76.6)	1.153 (1.053–1.253)	< 0.001	1.091 (0.997–1.194)	0.057	1.158 (1.066–1.258)	< 0.001
Race							
White	4802 (66.6)	1 (Reference)		1 (Reference)		1 (Reference)	
Black	981 (13.6)	1.128 (1.204–1.243)	0.015	1.033 (0.931–1.148)	0.540	0.862 (0.782–0.951)	0.003
Other	1428 (19.8)	0.835 (0.763–0.914)	< 0.001	0.894 (0.809–0.988)	0.028	0.679 (0.602–0.767)	< 0.001
Marital status							
Married	5674 (78.7)	1 (Reference)					
Unmarried	1537 (21.3)	1.017 (0.937–1.097)	0.680				
Grade							
Grade I–II	5581 (77.4)	1 (Reference)		1 (Reference)		1 (Reference)	
Grade III–IV	1630 (22.6)	1.663 (1.542–1.784)	< 0.001	1.116 (1.024–1.217)	0.013	1.904 (1.764–2.054)	< 0.001
T stage							
T1	3325 (46.1)	1 (Reference)		1 (Reference)		1 (Reference)	
T2	1756 (24.4)	1.257 (1.147–1.367)	< 0.001	1.309 (1.184–1.447)	< 0.001	1.272 (1.159–1.397)	< 0.001
T3	1853 (25.7)	3.012 (2.781–3.243)	< 0.001	1.307 (1.185–1.442)	< 0.001	3.852 (3.549–4.180)	< 0.001
T4	277 (3.8)	3.261 (2.753–3.769)	< 0.001	1.430 (1.193–1.174)	< 0.001	4.221 (3.615–4.929)	< 0.001
N stage							
N0	6717 (93.1)	1 (Reference)		1 (Reference)			
N1	494 (6.9)	2.468 (2.148–2.788)	< 0.001	1.031 (0.902–1.179)	0.650		
M stage							
M0	6474 (89.8)	1 (Reference)		1 (Reference)			
M1	737 (10.2)	2.900 (2.620–3.180)	< 0.001	0.916 (0.702–1.194)	0.520		
Surgery							
No surgery	3734 (51.8)	1 (Reference)		1 (Reference)		1 (Reference)	
Destruction	766 (10.6)	0.337 (0.298–0.380)	< 0.001	0.732 (0.634–0.845)	< 0.001	4.016 (3.526–4.575)	< 0.001
Resection	1118 (15.5)	0.278 (0.249–0.311)	< 0.001	0.498 (0.430–0.576)	< 0.001	0.784 (0.664–0.926)	< 0.001
Lobectomy	793 (11.0)	0.368 (0.326–0.416)	< 0.001	0.559 (0.481–0.651)	< 0.001	1.049 (0.884–1.245)	0.582
Hepatectomy	787 (10.9)	0.059 (0.046–0.078)	< 0.001	0.160 (0.119–0.215)	0.120	0.158 (0.117–0.212)	< 0.001
Transplant	13 (0.2)	0.145 (0.040–0.250)	< 0.001	0.313 (0.073–1.341)	0.660	0.382 (0.095–1.536)	0.175
Surgery to LN							
Yes	574 (8.0)	1 (Reference)		1 (Reference)			
No	6637 (92.0)	2.683 (2.258–3.108)	< 0.001	0.958 (0.789–1.163)	0.660		
Radiation							
Yes	707 (9.8)	1 (Reference)		1 (Reference)		1 (Reference)	
No	6504 (90.2)	0.691 (0.628–0.754)	< 0.001	1.171 (1.053–1.302)	< 0.001	1.502 (1.354–1.666)	< 0.001
Chemotherapy							
Yes	2613 (36.2)	1 (Reference)		1 (Reference)		1 (Reference)	
No	4598 (63.8)	0.722 (0.676–0.768)	< 0.001	0.907 (0.838–0.983)	0.017	1.353 (1.263–1.450)	< 0.001
AFP							
Negative	1949 (27.0)	1 (Reference)		1 (Reference)		1 (Reference)	
Positive	3903 (54.1)	1.752 (1.614–1.890)	< 0.001	1.306 (1.188–1.435)	< 0.001	1.877 (1.721–2.048)	< 0.001
Unknown	1359 (18.8)	1.366 (1.223–1.509)	< 0.001	1.210 (1.074–1.363)	< 0.001	1.477 (1.323–1.649)	< 0.001
Tumor size (mm)							
≤ 41	3060 (42.4)	1 (Reference)		1 (Reference)		1 (Reference)	
42–65	1595 (22.1)	2.056 (1.872–2.240)	< 0.001	1.358 (1.220–1.510)	< 0.001	2.198 (1.995–2.421)	< 0.001
≥ 66	2566 (35.4)	3.317 (3.056–3.578)	< 0.001	1.550 (1.389–1.729)	< 0.001	4.037 (3.714–4.387)	< 0.001
Bone metastasis							
Yes	180 (2.5)	1 (Reference)		1 (Reference)			
No	7031 (97.5)	0.377 (0.315–0.439)	< 0.001	0.956 (0.764–1.196)	0.690		
Brain metastasis							
Yes	21 (0.3)	1 (Reference)		1 (Reference)			
No	7190 (99.7)	0.325 (0.196–0.454)	< 0.001	0.814 (0.471–1.404)	0.458		
Liver metastasis							
Yes	50 (0.7)	1 (Reference)		1 (Reference)			
No	7161 (99.3)	0.412 (0.272–0.553)	< 0.001	1.280 (0.843–1.943)	0.253		
Lung metastasis							
Yes	294 (4.1)	1 (Reference)		1 (Reference)			
No	6917 (95.9)	0.394 (0.334–0.454)	< 0.001	1.115 (0.907–1.371)	0.297		
Distant LN metastasis							
Yes	595 (8.3)	1 ( Reference)		1 (Reference)		1 (Reference)	
No	6616 (91.7)	0.344 (0.308–0.380)	< 0.001	0.757 (0.586–0.977)	0.033	4.613 (4.162–5.113)	< 0.001

### Development and validation of the prognostic nomograms

**Fig. 5:**
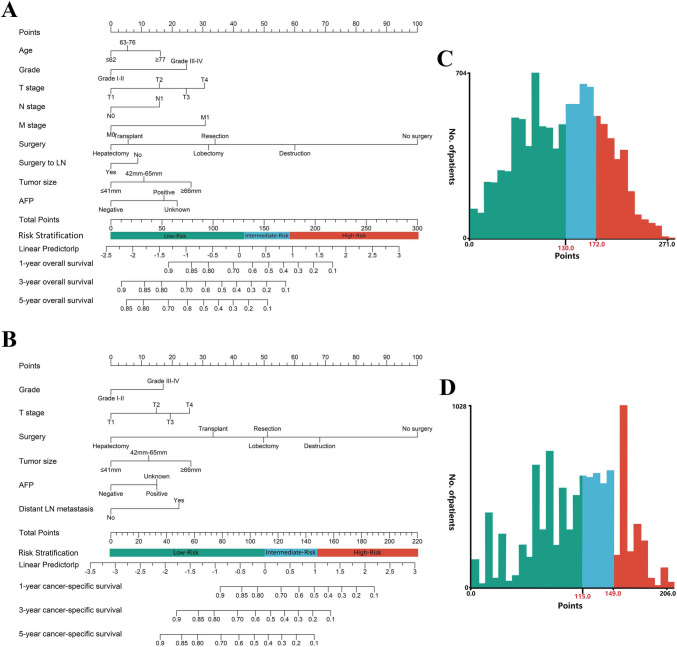
Nomograms and risk stratification model. **A** Nomogram predicting 1-, 3-, and 5-year overall survival. **B** Nomogram predicting 1-, 3-, and 5-year cancer-specific survival. **C** Risk stratification model based on the overall survival nomogram. **D** Risk stratification model based on the cancer-specific survival nomogram

According to the above screening results, age, grade, T stage, N stage, M stage, surgery, surgery to LN, AFP, and tumor size were selected to construct the OS prognostic nomogram (Fig. 5A), while grade, T stage, surgery, AFP, tumor size, and distant LN metastasis were selected to construct the CSS prognostic nomogram (Fig. 5B). Based on the X-tile software, the two best cutoff values for classifying the three prognostic risk groups were 130 and 179 (Fig. 5C). In addition to the risk classification system for OS, a risk classification system for CSS was also developed according to the total scores of each patient produced by the nomograms to divide all patients into three prognostic groups. Based on the X-tile software, the two best cutoff values for classifying the three prognostic risk groups were 115 and 149 (Fig. 5D). The nomogram scores corresponding to the OS and CSS at 1, 3, and 5 years were listed in Supplementary Table 2. The dynamic nomograms of OS (https://livercancernomogram.shinyapps.io/DynNomapp/) and CSS (https://cancer-specificsurvival.shinyapps.io/DynNomapp/) were developed to facilitate clinical application (Supplementary Fig. 2).

**Fig. 6:**
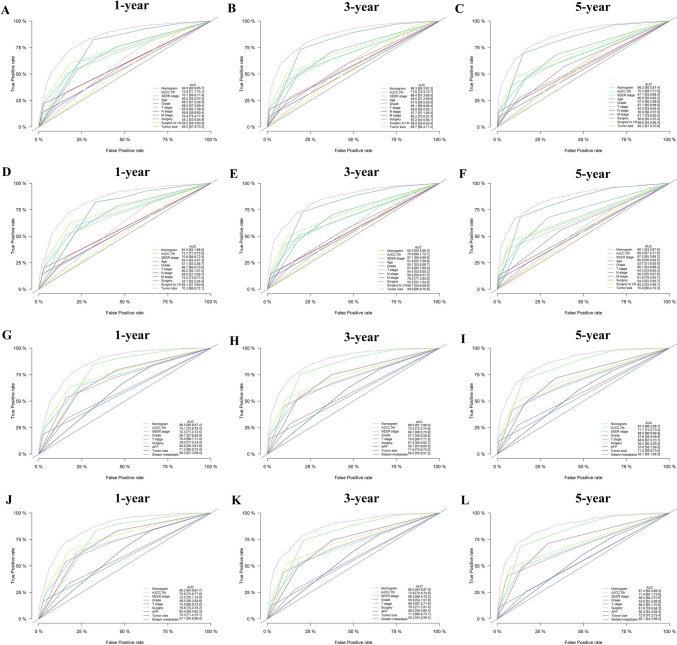
ROC curves of nomograms, AJCC staging, SEER staging, and individual independent variables in the training set and validating set. **A**–**C** For 1-, 3-, and 5-year overall survival (OS) in the training set; **D**–**F** For 1-, 3-, and 5-year overall survival (OS) in the validation set; **G**–**I** For 1-, 3-, and 5-year cancer-specific survival (CSS) in the training set; **J**–**L** For 1-, 3-, and 5-year cancer-specific survival (CSS) in the validation set

**Fig. 7:**
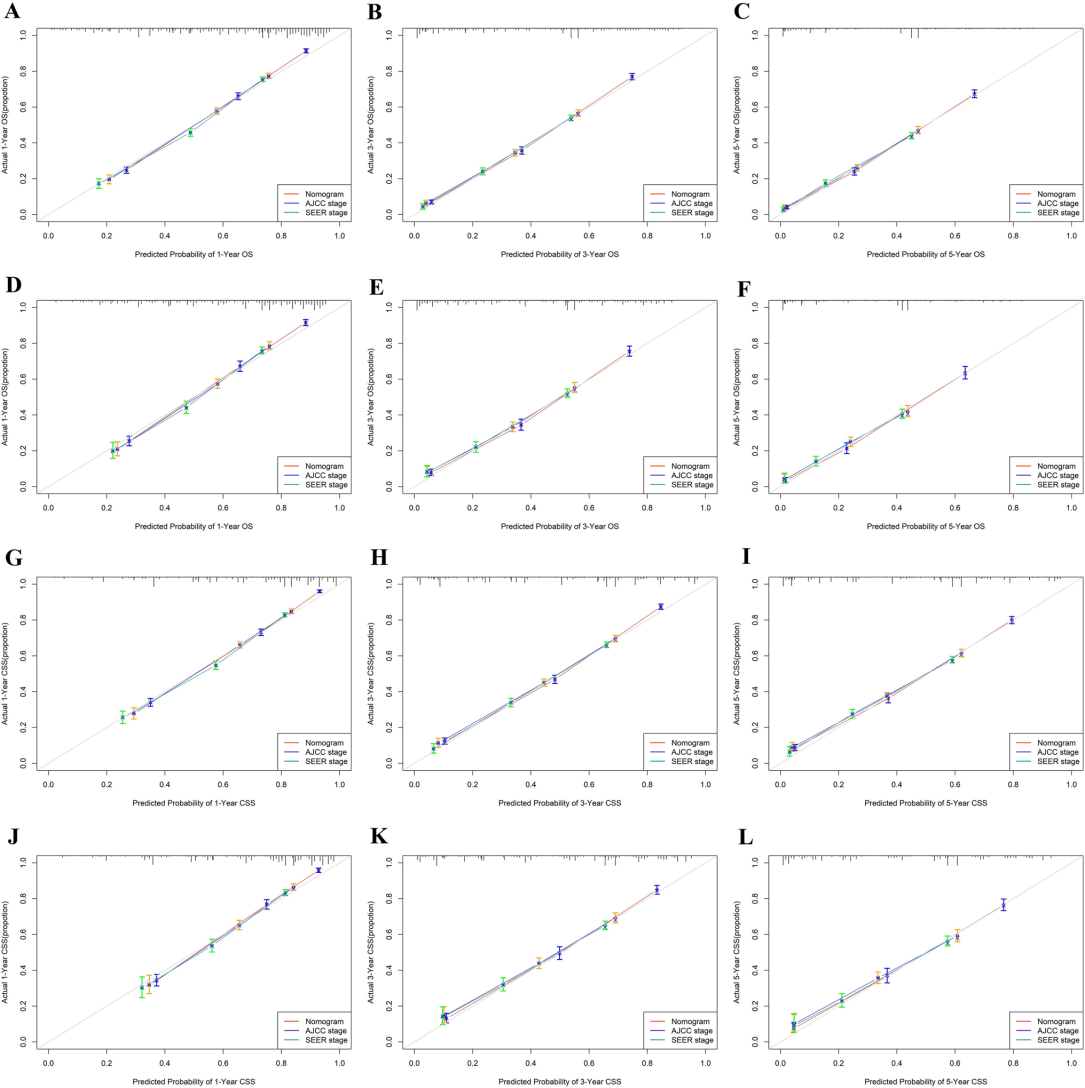
Calibration curves of the nomograms, AJCC staging, and SEER staging. **A**–**C** For 1-, 3-, and 5-year overall survival (OS) in the training set; **D**–**F** For 1-, 3-, and 5-year overall survival (OS) in the validation set; **G**–**I** For 1-, 3-, and 5-year cancer-specific survival (CSS) in the training set; **J**–**L** For 1-, 3-, and 5-year cancer-specific survival (CSS) in the validation set

**Fig. 8:**
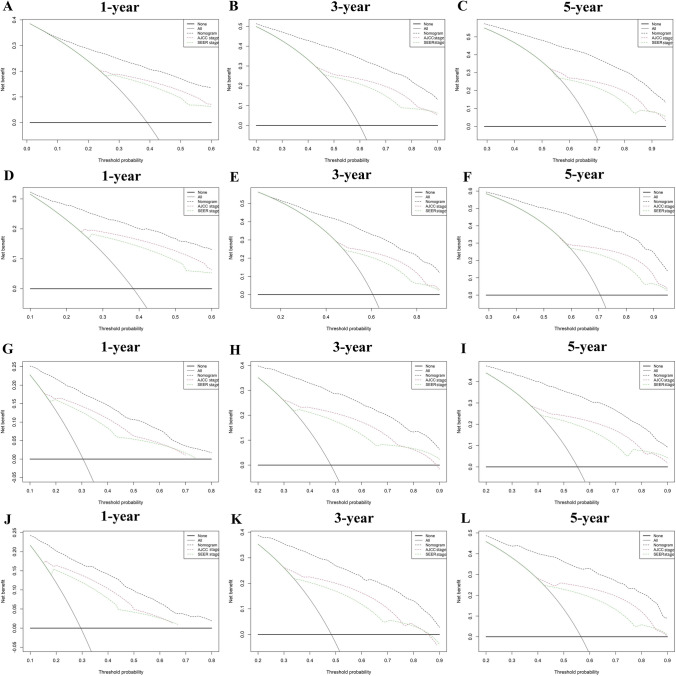
DCA curves of nomograms, AJCC staging, and SEER staging. **A**–**C** For 1-, 3-, and 5-year overall survival (OS) in the training set; **D**–**F** For 1-, 3-, and 5-year overall survival (OS) in the validation set; **G**–**I** For 1-, 3-, and 5-year cancer-specific survival (CSS) in the training set; **J**–**L** For 1-, 3-, and 5-year cancer-specific survival (CSS) in the validation set

In the training set, the C-index of the nomograms predicting 1-, 3-, and 5-year OS were 0.788, 0.792, and 0.790. The AUC of the nomograms predicting 1-, 3-, and 5-year OS were 0.848, 0.863, and 0.862. In the validation set, the C-index and AUC values had similar results (Supplementary Tables 3 and 4). For CSS, the C-index of the nomograms were 0.803, 0.808, and 0.806, and the AUC of the nomograms was 0.865, 0.880, and 0.874 in the training set. (Supplementary Tables 3 and 4). Similar results were also found in the validation set. Compared with the AJCC staging and SEER staging, the nomograms had higher C-index, time-dependent AUC, NRI, and IDI (Supplementary Tables 5 and 6). Additionally, the nomograms had lower AIC and BIC, which suggested better predictive performance with no significant overfitting (*P* < 0.001) (Supplementary Table 3). The ROC curve was greater than AJCC staging, suggesting the favorable discrimination of the nomograms (Fig. 6). The calibration curves indicated that the nomograms had a strong calibration in the training set and validation set (Fig. 7). DCA curves showed that the nomograms had better clinical benefits than AJCC staging and SEER staging (Fig. 8).

### Risk stratification for HCC patients

**Fig. 9:**
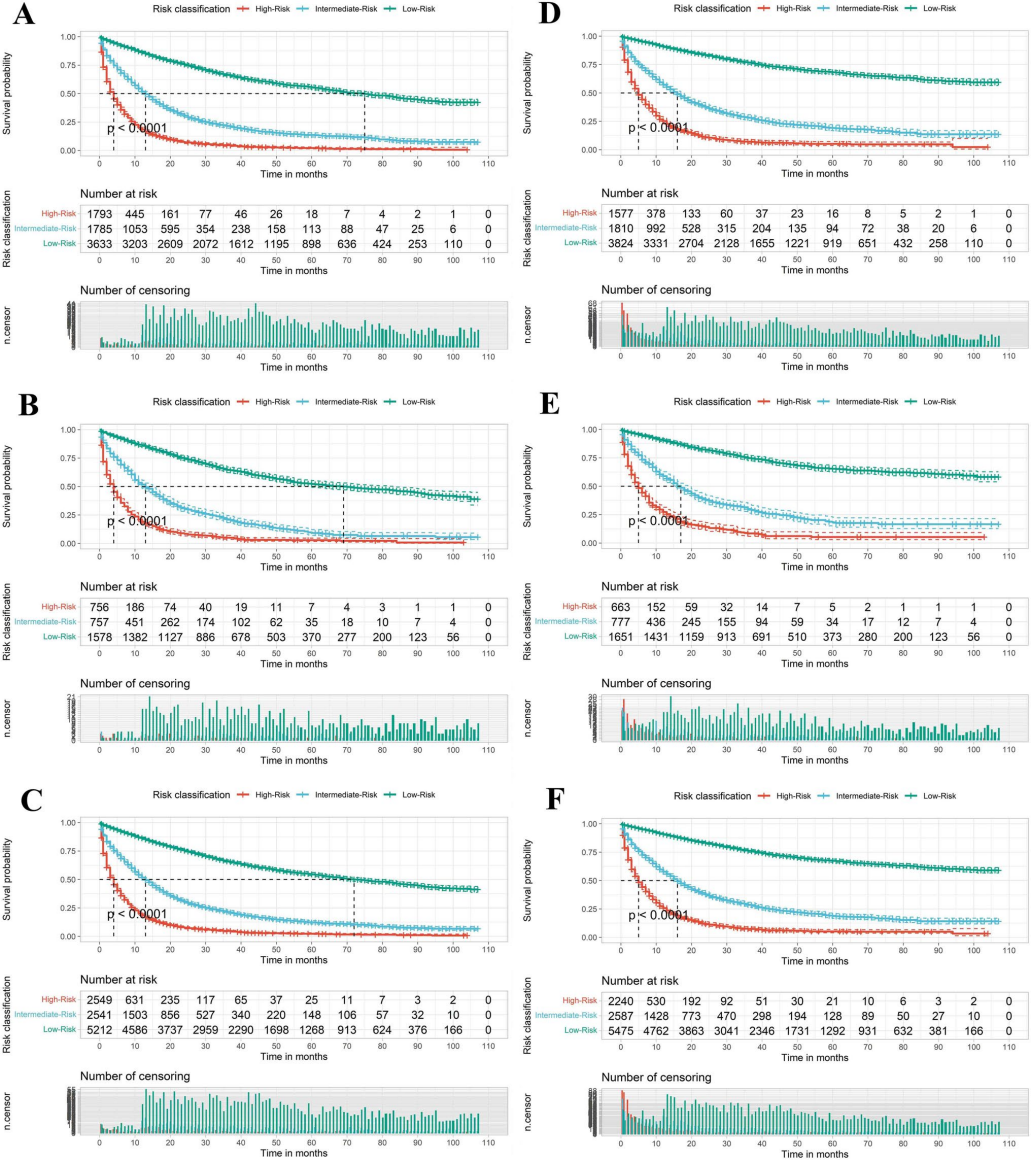
Survival curves showed the survival status classified by the overall survival (OS) nomogram of the training set (**A**), the validation set (**B**), and all patients (**C**) in primary hepatocellular carcinoma. Survival curves showed the survival status classified by the cancer-specific survival (CSS) nomogram of the training set (D), the validation set (**E**), and all patients (**F**) in primary hepatocellular carcinoma

A risk classification system for OS was developed according to the total scores of each patient produced by the nomograms to divide all patients into three prognostic groups. The results of the KM survival analysis with the log-rank test showed that there existed different OS and CSS in three risk groups of patients with HCC (Fig. 9A, D). The low-risk group had a better prognosis than the high-risk group (*P* < 0.001). Similar patterns were also observed within the validation cohort and total cohort (Fig. 9B–F). These results suggest that the nomograms have the potential to stratify HCC patients into three distinct prognostic groups. Furthermore, the oncological characteristics of the three risk groups exhibited differences. The high-risk group had a larger proportion of patients with T3, T4, N1, M1, high grade, and large tumor size compared to the low-risk group (Supplementary Fig. 3).

### Risk stratification for subgroup analysis

**Fig. 10:**
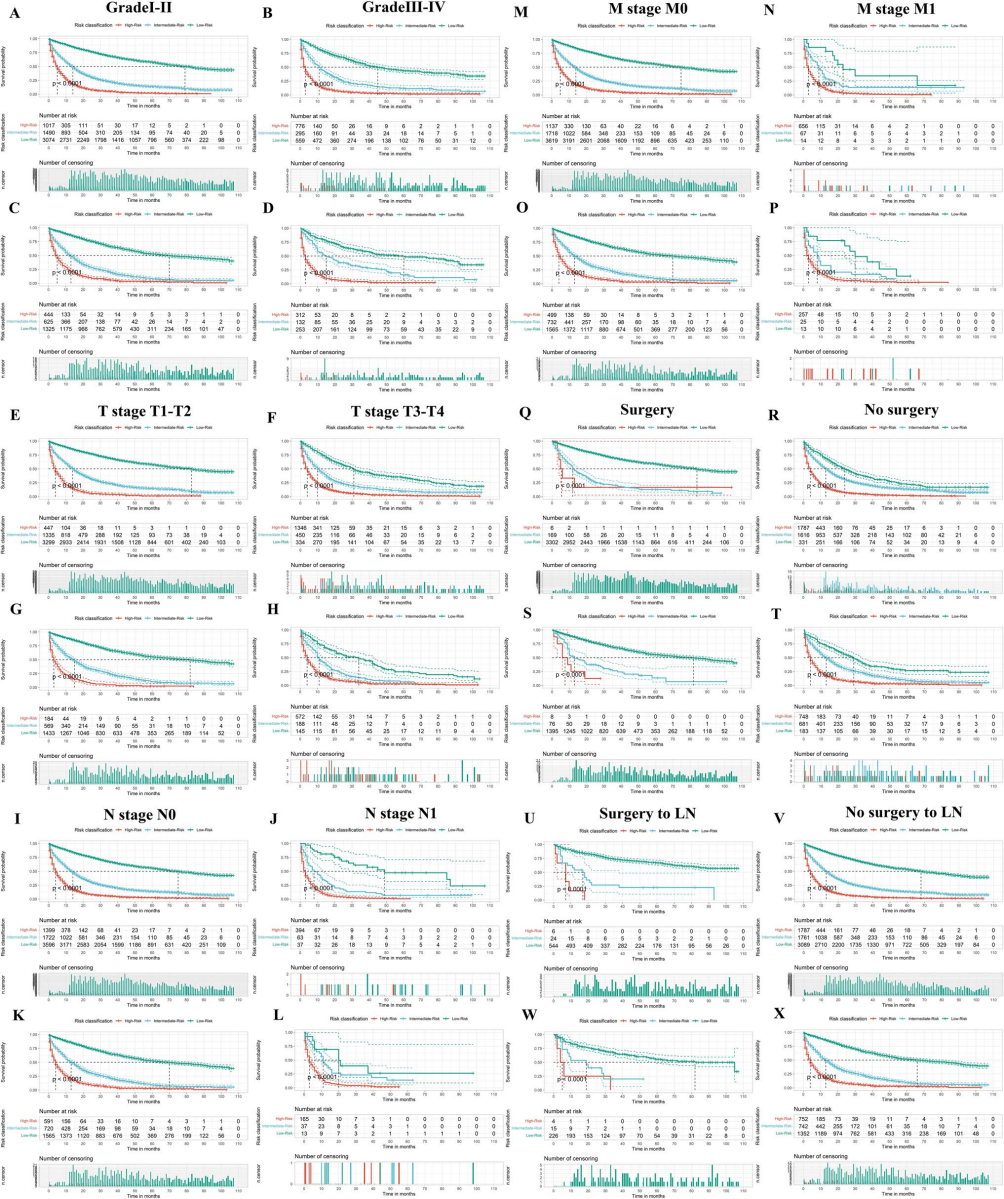
Subgroup analysis of OS stratification in the training set (**A**, **B**) and validation set (**C**, **D**) according to grade. Subgroup analysis of OS stratification in the training set (**E**, **F**) and validation set (**G**, **H**) according to T stage. Subgroup analysis of OS stratification in the training set (**I**, **J**) and validation set (**K**, **L**) according to N stage. Subgroup analysis of OS stratification in the training set (**M**, **N**) and validation set (**O**, **P**) according to M stage. Subgroup analysis of OS stratification in the training set (**Q**, **R**) and validation set (**S**, **T**) according to surgery. Subgroup analysis of OS stratification in the training set (**U**, **V**) and validation set (**W**, **X**) according to surgery to LN

HCC patients were divided into different subgroups based on age, grade, T stage, N stage, M stage, surgery, surgery to LN, AFP, and tumor size to evaluate the performance of the risk stratification system of OS. As shown in Fig. 10 and Supplementary Fig. 4, high-risk patients have a worse prognosis than low- and intermediate-risk patients in each subgroup. Similar results were found in the subgroup analysis of the CSS risk stratification system (Supplementary Fig. 5). The results of the subgroup analysis demonstrate that the nomograms have the potential to stratify HCC patients into different OS and CSS subgroups, which indicates that the nomograms are effective for distinguishing the prognosis in different HCC patient subgroups.

## Discussion

HCC is aggressive cancer that displays high molecular diversity and a propensity for postoperative relapse [2]. The survival outcomes of HCC patients are heavily influenced by complex tumor characteristics and the wide range of therapeutic modalities [3]. To date, few accurate and user-friendly models exist for predicting the prognosis of HCC. In this study, we developed clinical prognostic nomograms of HCC patients utilizing multiple clinicopathological variables obtained from the SEER database. The results of AIC, BIC, C-index, AUC, calibration curves, and DCA curves validated the robust discrimination and superior net benefit of our nomograms in the prognosis of HCC patients. According to the model, HCC patients could be effectively divided into three groups (high-, intermediate-, and low-risk groups) with significant OS and CSS. In addition, the results of the subgroup survival curves demonstrate that nomograms can provide reliable risk stratification of HCC patients.

In this study, we identified the independent prognostic factors for the OS and CSS of HCC patients. From the perspective of the patient’s condition, we found that age was one of the prognostic factors in HCC patients. Our survival analysis revealed that older age was significantly associated with shorter OS, but did not exhibit such a correlation with CSS. Cumulative survival diminishes with increase in patient age, and survival was inversely associated with age at diagnosis, which was in agreement with the results of a previous retrospective study [23]. The potential reason for the reduced survival rate in elderly HCC patients may be the cumulative impact of liver damage due to chronic liver disease, as well as other risk factors such as obesity and diabetes, which are commonly encountered in the elderly population [24].

From the perspective of the tumor, tumor size, T stage, N stage, M stage, histological grade, distant lymph node metastasis, and AFP level were identified as independent prognostic factors of HCC. Tumor size, tumor stage, and degree of differentiation are closely related to the biological behavior of the malignant tumor. Generally, larger tumors tend to result in worse clinical outcomes, higher risk of recurrence, and increased mortality than smaller ones [25]. In addition, tumor size is also an important factor to select the treatment for HCC [26]. Small tumors may be more effectively treated with curative treatments such as surgery, and radiofrequency ablation, while large tumors may require more intensive treatments such as transarterial chemoembolization (TACE), systemic chemotherapy, or radiation therapy [27]. Our study further confirmed that tumor size is an independent prognostic factor for HCC patients, which may provide a useful reference in predicting mortality risk and selecting appropriate treatment. The histological grade is also crucial in assessing the aggressiveness of HCC, selecting treatment options, and predicting outcomes [28]. HCCs are typically graded on a scale of 1–4, with a higher grade indicating a more aggressive neoplasm. Previous studies have shown a strong correlation between high histological grade and poor survival [29]. Our study verified the prognostic value of histological grade for HCC, which facilitates the assessment of the aggressiveness of cancer. Regarding distant lymph node metastasis, it is recognized as an important route of HCC dissemination, thereby serving as an important marker of invasiveness [30]. Previous studies have indicated that HCC patients with lymph node metastasis have worse prognoses compared to those without metastasis [31]. Early management of lymph nodes has the potential to prolong survival [30]. Our survival analysis further validated that distant lymph node metastasis is an independent prognostic factor for HCC patients, which may guide early intervention. AFP, a glycoprotein, has been widely utilized as a diagnostic and prognostic biomarker in HCC patients [32]. Elevated AFP levels correlate with larger tumor sizes and poorer prognosis [33]. Consistent with previous observations, our findings suggest that AFP is a prognostic factor in HCC and that increased AFP levels are negatively associated with both overall survival and cancer-specific survival. The T stage denotes the magnitude and extent of the primary tumor in the liver. The T stage (tumor size) has consistently been regarded as a vital prognostic factor for HCC and has been extensively incorporated in various conventional HCC staging systems for guiding therapy [34]. For instance, early-stage HCC may be eligible for surgery, while advanced-stage HCC may necessitate systemic therapy [35]. Vascular invasion of multiple tumors in hepatocellular carcinoma (HCC) may herald advanced T stage. The vascular invasion has the potential to promote the spread of malignant cells to distant organs via the bloodstream, thereby enhancing tumor growth and metastasis [36]. Metastatic disease (including lymph node metastasis and distant metastasis) was regarded as a sign of advanced stage [37]. The presence of metastases is associated with a worse outcome than HCC without metastases. One reason for the difference in outcome is that metastatic disease often implies that cancer has extended beyond the liver and is affecting other vital organs, such as the lungs or bones [12]. This can make treatment more difficult and may limit the options available. In addition, metastatic HCC is more likely to be associated with underlying liver dysfunction, such as cirrhosis, which can further complicate treatment and contribute to a poor outcome. Another factor that may explain the worse outcome of metastatic HCC is that it tends to be less responsive to treatment than localized HCC [38]. We included T, N, and M stages in the nomograms and found that risk scores were higher for T3–T4, N1, and M1 stages than for T1–T2, N0, and M0 stages, indicating a worse prognosis.

From the perspective of therapies, resection, lobectomy, hepatectomy, transplant, and surgery to lymph nodes were independent favorable factors for HCC patients. These methods had a superior ability to improve the prognosis of HCC patients compared to no treatment. Furthermore, patients who had liver resection had a better prognosis, followed by liver transplantation, confirming the previous finding [39–41]. Surgical resection seems to be the optimal treatment strategy for HCC, especially for early-stage patients [42]. Additionally, the application of lymph node surgery can also improve the prognosis of HCC patients, which should be related to reducing tumor distant metastasis [43]. It is noteworthy that adjuvant therapies, such as chemotherapy and radiotherapy, are usually deemed to prolong the survival of cancer patients [44, 45]. However, there has been some controversy surrounding this idea. A meta-analysis indicated that fluorouracil-based adjuvant chemotherapy does not improve overall survival in patients with colorectal cancer [46]. So far, severe lymphocyte depletion induced by radiotherapy was an unfavorable prognostic factor for overall survival in lung cancer patients [47]. In our study, radiotherapy and chemotherapy were also not identified as independent prognostic factors for HCC patients. Therefore, the benefit of chemotherapy and radiotherapy in HCC patients still needs further investigation.

Compared with previous studies, this study made the following improvements. Firstly, subgroup analysis results manifested that our nomograms had high accuracy in each subgroup, such as in predicting the prognosis of AFP-positive and elderly HCC [48, 49]. Second, we employed the competing risk model and the LASSO method to select the prognostic factors. The competing risk model offers a solution to the limitation of the Cox risk model, which is typically employed in etiological studies, as it allows for the simultaneous and more accurate consideration of multiple endpoint events. Moreover, the LASSO regression can address the issue of overfitting [50]. Thirdly, our study selected patients with AFP tests during 2010–2017 for analysis [51]. We found that AFP was indeed an important prognostic factor for HCC, providing higher predictive accuracy. Fourthly, we employed several novel indicators to assess the performance of our study, including C-index, AUC, NRI, IDI, AIC, and BIC. These indicators provided compelling evidence that our model is excellent in predicting the prognosis of patients with hepatocellular carcinoma. Finally, liver resection and liver transplantation are important current treatments for HCC [50], and liver resection can be divided into lobectomy and hepatectomy, with patient prognostic outcomes likely to vary depending on the surgery chosen. If the information on surgery is dichotomized, then the impact of different surgical approaches on the prognosis of HCC patients cannot be studied. Our study divided surgical treatment into multiple variables, but not a binary variable. We examined the impact of different treatment approaches on HCC and obtained a more comprehensive prognostic analysis. Our developed nomograms improve on the inherent deficiencies of AJCC staging by incorporating several important HCC risk factors such as age, grade, and AFP [52]. In addition, the developed nomograms can stratify risk compared to AJCC staging. Tumor stratification may enable clinicians to devise tailored therapeutic approaches to achieve improved clinical outcomes for patients.

Our study is based on SEER data [13]. Owing to big data, the diagnosis of patients is precisely categorized, eliminating the interference of their malignant tumor history. Moreover, the number of HCC patients recorded in the SEER database is immense, which facilitates us to construct a more accurate model. In addition, the items incorporated in our nomograms are common clinically, easily accessible, and comprehensible items that can be easily implemented even in primary hospitals.

This study represented one of the largest cohorts focusing on the prognosis of HCC patients. The data were collected from multiple centers, and heterogeneity in various centers could be successfully resolved. However, our study has some limitations. First, this large-sample retrospective study was based on the SEER database, which may have some inherent biases. Second, data regarding several potentially important prognosis-related factors such as microvascular invasion, hepatitis status, performance status, Child score, MELD score, and anti-viral therapy were not available in the SEER database. Third, the predictive model was developed based on data obtained from the SEER database, which cannot represent the global population. Although our nomograms did not integrate all the prognostic factors mentioned above, they still achieved a relatively specific prediction of the prognosis of HCC patients and had a significantly higher C-index than the conventional staging systems. Our nomograms were internally validated, and it needs to be validated externally using other populations.

## Conclusion

In this study, we screened out the independent factors of OS and CSS in HCC patients. Incorporating the identified indicators, we constructed and validated the nomograms to predict the prognosis of OS and CSS. The models showed superior prediction ability, which may enable clinicians to obtain personal predictive information to ascertain whether a patient is at high risk of death. Our study would contribute to the effective management of HCC patients and the improvement of their quality of life.

## Supplementary Information

Below is the link to the electronic supplementary material.

Supplementary file1 (DOCX 6950 KB)

Supplementary file2 (DOCX 53 KB)

## Data Availability

Publicly available datasets were analyzed in this study. A total of 10,302 cases of available providing patient information and clinical characteristics were obtained from the SEER database during the period of 2010 to 2017. The SEER ∗ Stat software (version 8.3.9, National Cancer Institute, Bethesda, MD, USA) was utilized to extract patient data with complete follow-up from the SEER database.
