# Supplementary material for: Development and evaluation of nomograms and risk stratification systems to predict the overall survival and cancer-specific survival of patients with hepatocellular carcinoma
Source: Clin Exp Med. 2024 Feb 28;24(1):44. doi: 10.1007/s10238-024-01296-1 (PMC10899391; doi:10.1007/s10238-024-01296-1)
Supplement: Supplementary file 1 — Supplementary file1 (DOCX 6950 KB) [file 10238_2024_1296_MOESM1_ESM.docx]

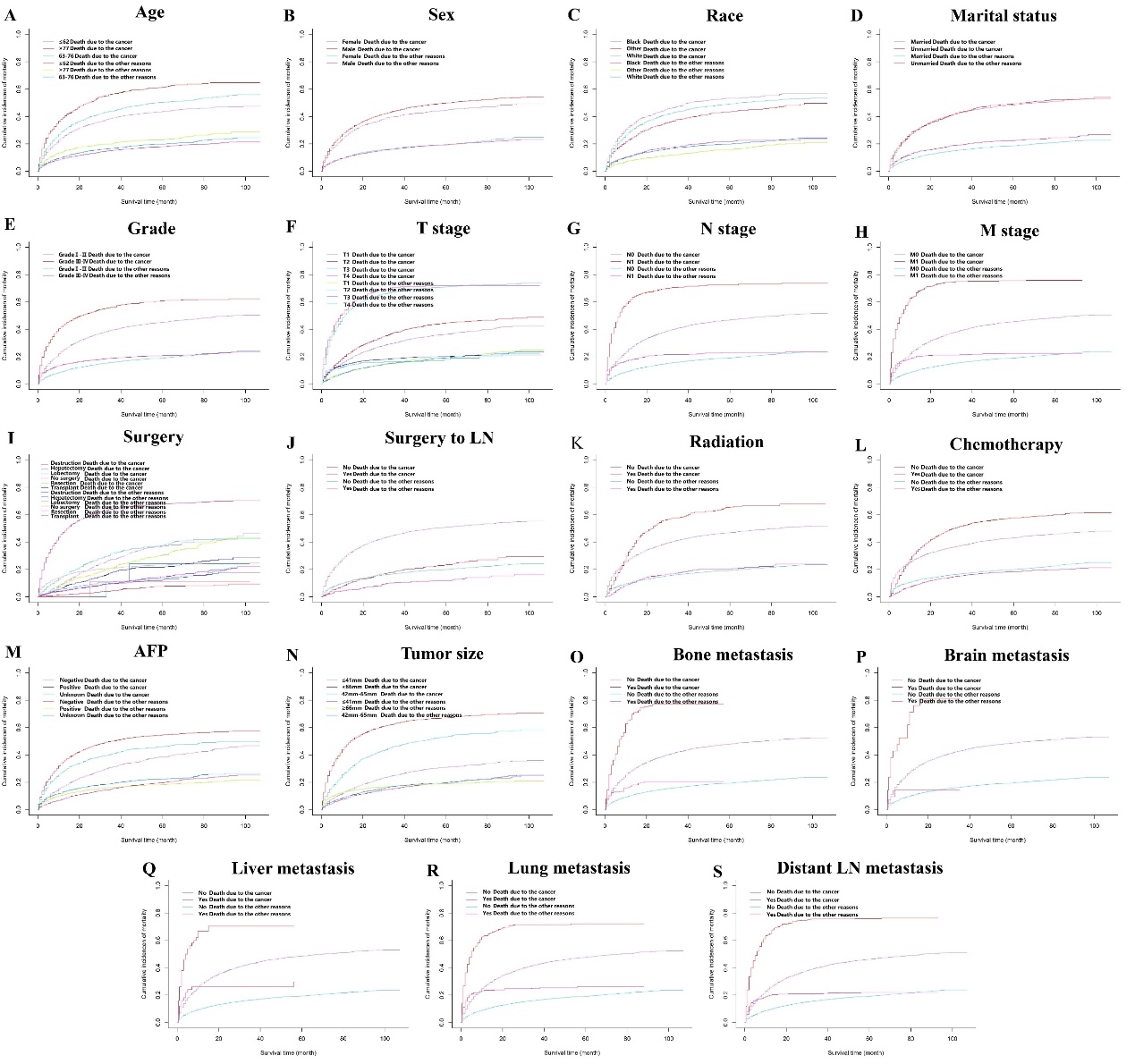


**Supplementary Figure 1.** Competing risk analyses for patients in the training cohort according to age (A), sex (B), race (C), marital status (D), grade (E), T stage (F), N stage (G), M stage (H), surgery (I), surgery to lymph node (J), radiation (K), chemotherapy (L), AFP (M), tumor size (N), bone metastasis (O), brain metastasis (P), liver metastasis (Q), lung metastasis (R), distant lymph node metastasis (S).


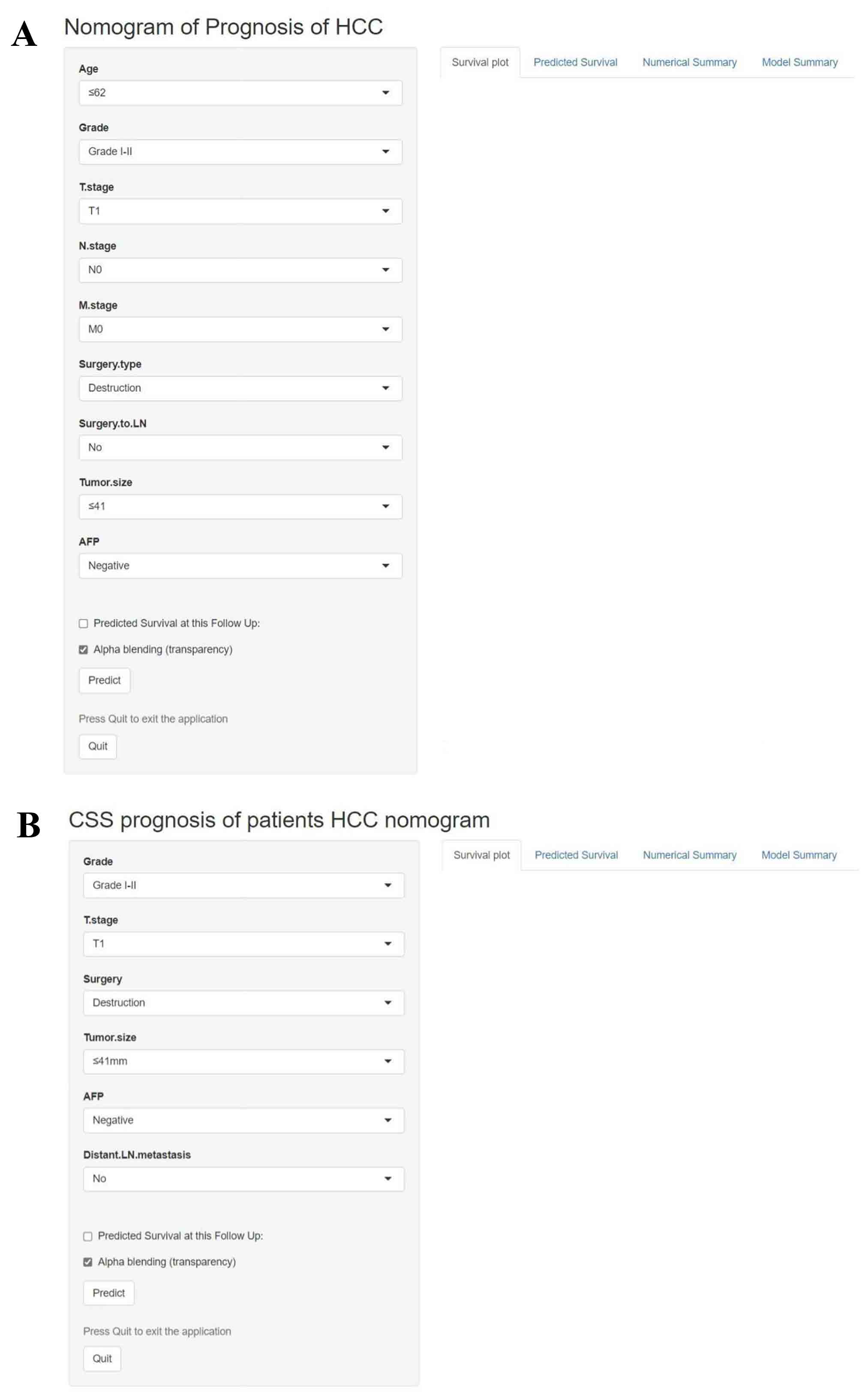


**Supplementary Figure 2.** Dynamic nomogram of overall survival (OS) (A) and cancer-specific survival (CSS) (B).


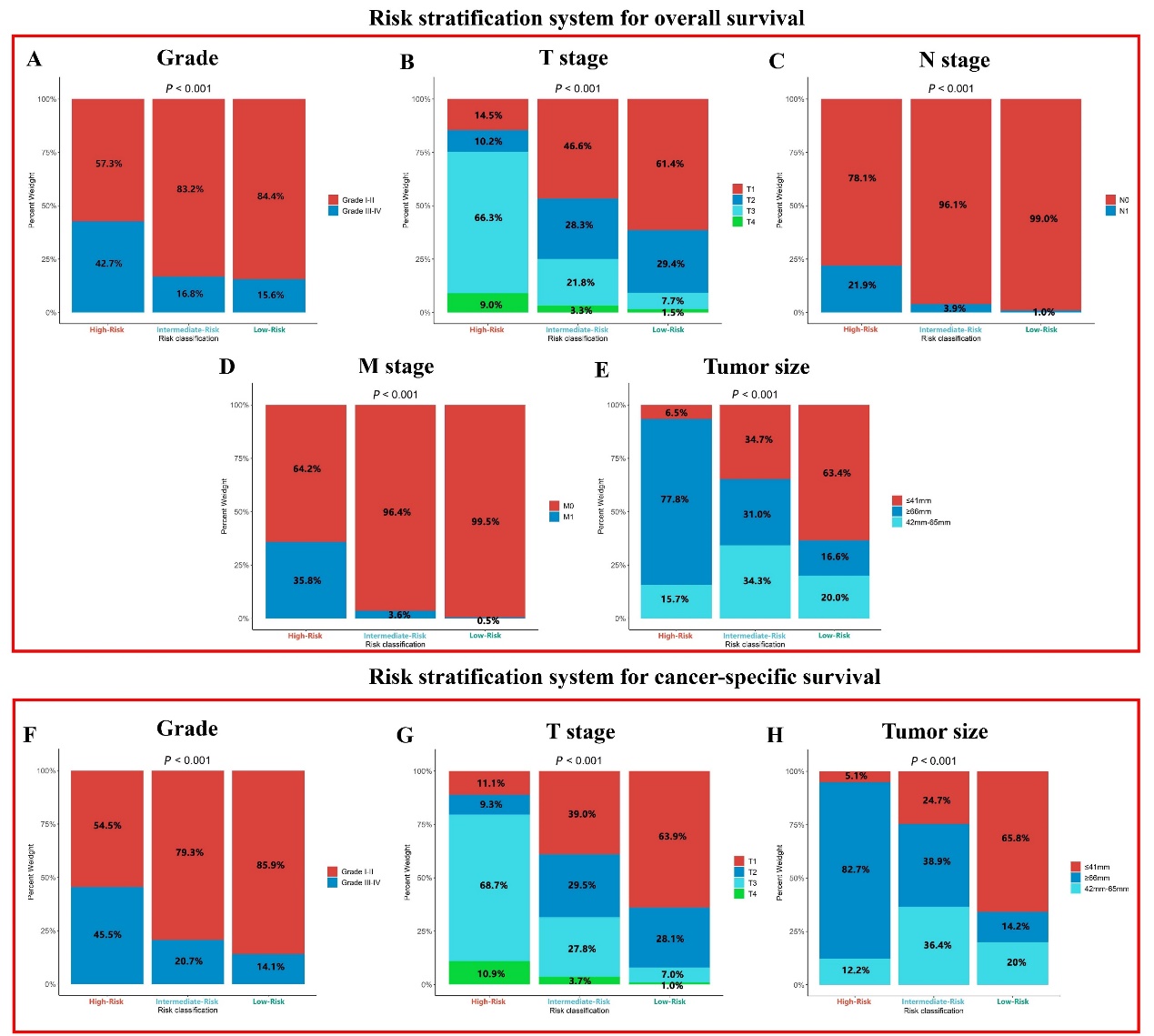


**Supplementary Figure 3.** Comparison of oncology features in nomograms of patients with different risk stratification. grade (A), T stage (B), N stage (C), M stage (D), and tumor size (E) distribution in patients with different risk stratification based on overall survival (OS) risk stratification (all *P* < 0.001). grade (F), T stage (J), and tumor size (K) distribution in patients with different risk stratification based on cancer-specific survival (CSS) risk stratification (all *P* < 0.001).


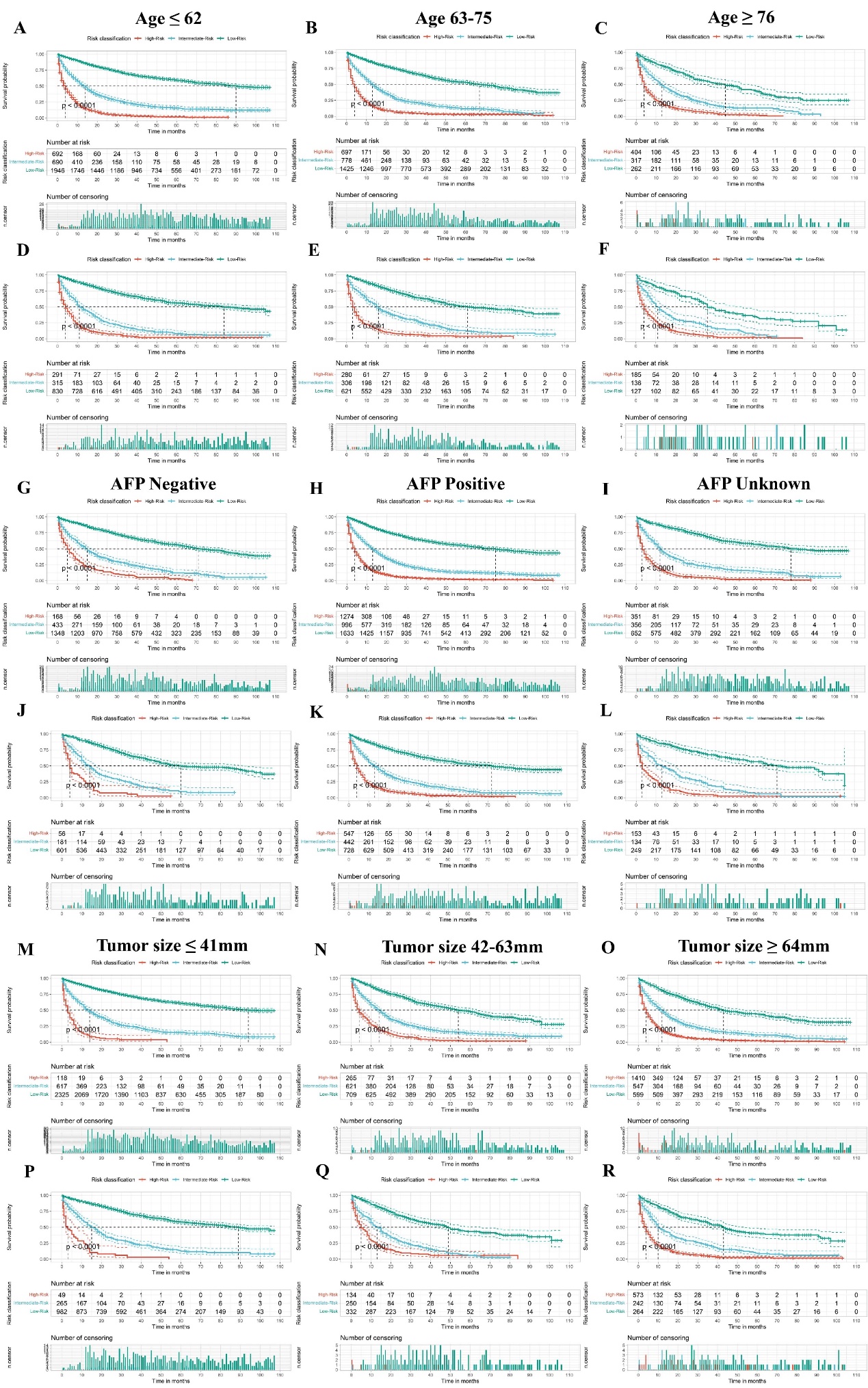


**Supplementary Figure 4.** Subgroup analysis of OS stratification in the training set (A-C) and validation set (D-F) according to age. Subgroup analysis of OS stratification in the training set (G-I) and validation set (J-L) according to AFP. Subgroup analysis of OS stratification in the training set (M-O) and validation set (P-R) according to tumor size.

**
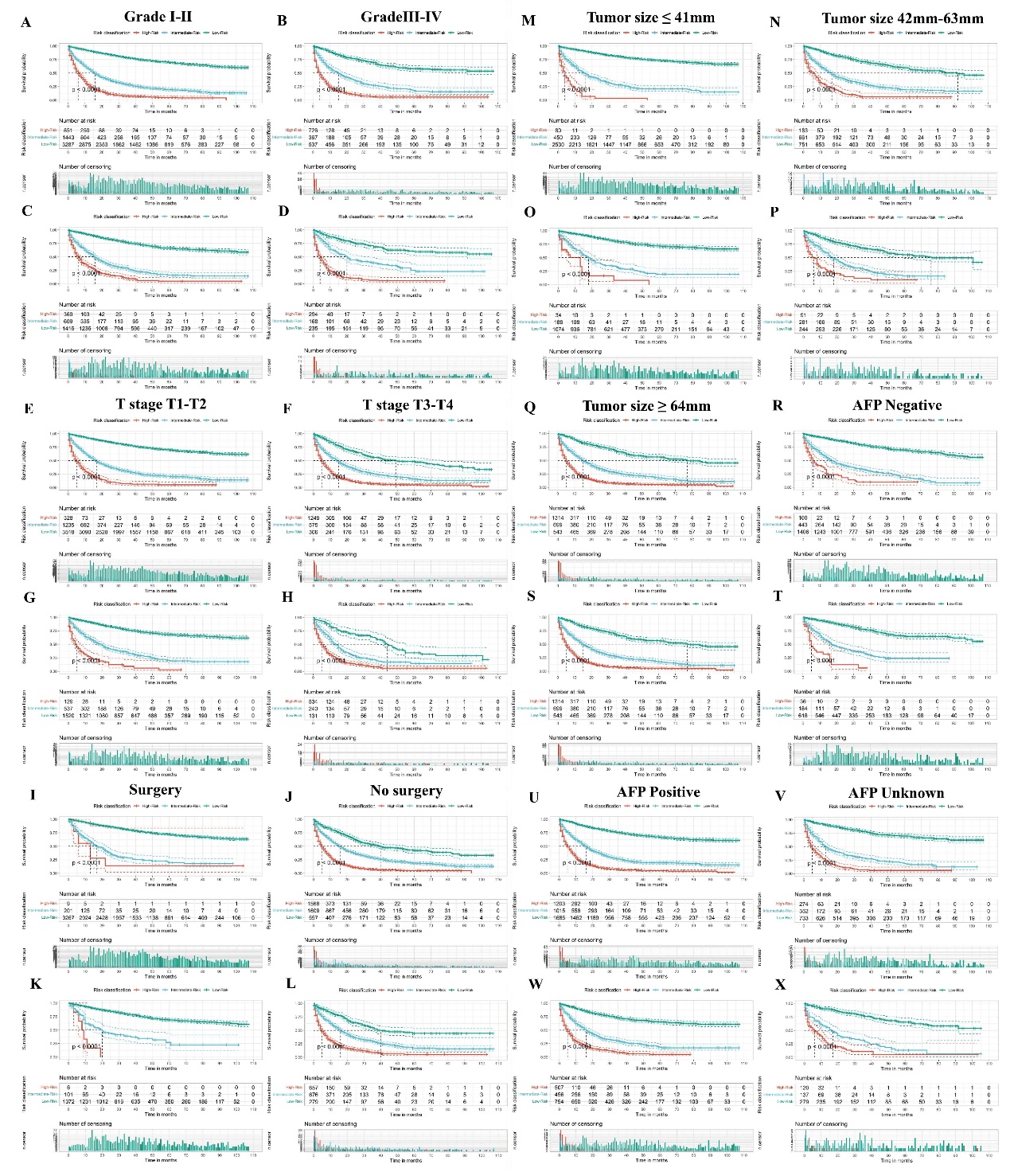
**

**Supplementary Figure 5.** Subgroup analysis of CSS risk stratification in the training set (A, B) and validation set (C, D) according to AFP. Subgroup analysis of CSS risk stratification in the training set(E, F) and validation set (G, H) according to T stage. Subgroup analysis CSS risk stratification in the training set(I, J) and validation set (K, L) according to surgery. Subgroup analysis of CSS risk stratification in the training set (M, N, O) and validation set (O, P, S) according to tumor size. Subgroup analysis of CSS risk stratification in the training set (R, U, V) and validation set (T, W, X) according to AFP.
