# Supplementary material for: Development and evaluation of nomograms and risk stratification systems to predict the overall survival and cancer-specific survival of patients with hepatocellular carcinoma
Source: Clin Exp Med. 2024 Feb 28;24(1):44. doi: 10.1007/s10238-024-01296-1 (PMC10899391; doi:10.1007/s10238-024-01296-1)
Supplement: Supplementary file 2 — Supplementary file2 (DOCX 53 KB) [file 10238_2024_1296_MOESM2_ESM.docx]

| Supplementary Table 1 Kaplan-Meier univariate analysis results and 1-,3-, 5-year OS and median survival time. | | | | | | | |
| --- | --- | --- | --- | --- | --- | --- | --- |
| Variable | **Number of patients, n (%)** | **Median survival** | **1-year OS** | **3-year OS** | **5-year OS** | **χ2** | ***P* value** |
| All patients | 7211 (100) | 22.0 (20.6-23.4) | 0.609±0.006 | 0.401±0.006 | 0.320±0.006 |  |  |
| Age | | | | | | 246.0 | ＜0.001 |
| ≤62 | 3328 (46.2) | 30.0 (27.3-32.7) | 0.657±0.008 | 0.458±0.090 | 0.384±0.090 |  |  |
| 63-76 | 2900 (40.2) | 20.0 (18.1-21.9) | 0.601±0.009 | 0.392±0.010 | 0.298±0.010 |  |  |
| ≥77 | 983 (13.6) | 11.0 (9.5-12.5) | 0.467±0.016 | 0.232±0.014 | 0.154±0.014 |  |  |
| Sex | | | | | | 9.5 | 0.002 |
| Female | 1687 (23.4) | 24.0 (20.8-27.2) | 0.631±0.012 | 0.431±0.013 | 0.357±0.013 |  |  |
| Male | 5524 (76.6) | 21.0 (19.6-22.4) | 0.602±0.007 | 0.392±0.007 | 0.308±0.007 |  |  |
| Race | | | | | | 69.5 | ＜0.001 |
| White | 6843 (66.4) | 20.0 (18.5-21.5) | 0.597±0.007 | 0.387±0.007 | 0.308±0.008 |  |  |
| Black | 1411 (13.7) | 16.0 (13.6-18.4) | 0.555±0.016 | 0.338±0.016 | 0.249±0.016 |  |  |
| Other | 2048 (19.9) | 36.0 (30.9-41.1) | 0.688±0.012 | 0.495±0.014 | 0.409±0.015 |  |  |
| Marital status | | | | | | 12.6 | ＜0.001 |
| Married | 5674 (78.7) | 22.0 (20.5-23.5) | 0.618±0.006 | 0.410±0.007 | 0.330±0.007 |  |  |
| Unmarried | 1537 (21.3) | 19.0 (16.4-21.6) | 0.573±0.013 | 0.367±0.013 | 0.282±0.013 |  |  |
| Grade | | | | | | 325.1 | ＜0.001 |
| Grade I-II | 5581 (77.4) | 28.0 (26.1-29.9) | 0.661±0.006 | 0.445±0.007 | 0.359±0.007 |  |  |
| Grade III-IV | 1630 (22.6) | 9.0 (8.0-10.0) | 0.429±0.012 | 0.249±0.011 | 0.186±0.011 |  |  |
| T stage | | | | | | 1477.6 | ＜0.001 |
| T1 | 3325 (46.1) | 43.0 (38.6-47.4) | 0.740±0.008 | 0.537±0.009 | 0.445±0.010 |  |  |
| T2 | 1756 (24.4) | 31.0 (27.7-34.3) | 0.695±0.011 | 0.466±0.013 | 0.361±0.013 |  |  |
| T3 | 1853 (25.7) | 6.0 (5.4-6.6) | 0.335±0.011 | 0.135±0.008 | 0.088±0.008 |  |  |
| T4 | 277 (3.8) | 6.0 (4.5-7.5) | 0.321±0.028 | 0.128±0.022 | 0.099±0.021 |  |  |
| N stage | | | | | | 616.8 | ＜0.001 |
| N0 | 6717 (93.1) | 25.0 (23.5-26.5) | 0.638±0.006 | 0.423±0.013 | 0.340±0.007 |  |  |
| N1 | 494 (6.9) | 4.0 (3.3-4.7) | 0.208±0.018 | 0.077±0.013 | 0.045±0.011 |  |  |
| M stage | | | | | | 1351.0 | ＜0.001 |
| M0 | 6474 (89.8) | 28.0 (26.3-29.7) | 0.660±0.006 | 0.442±0.007 | 0.353±0.007 |  |  |
| M1 | 737 (10.2) | 3.0 (2.5-3.5) | 0.163±0.014 | 0.036±0.008 | 0.022±0.006 |  |  |
| Surgery | | | | | | 2783.2 | ＜0.001 |
| No surgery | 3734 (51.8) | 8.0 (7.5-8.5) | 0.376±0.008 | 0.149±0.006 | 0.089±0.006 |  |  |
| Destruction | 766 (10.6) | 42.0 (37.2-43.8) | 0.835±0.013 | 0.542±0.019 | 0.412±0.021 |  |  |
| Resection | 1118 (15.5) | 66.0 (59.8-72.2) | 0.862±0.010 | 0.687±0.015 | 0.546±0.018 |  |  |
| Lobectomy | 793 (11.0) | 60.0 (49.6-70.4) | 0.802±0.014 | 0.586±0.019 | 0.500±0.021 |  |  |
| Hepatectomy | 787 (10.9) | —— | 0.929±0.009 | 0.845±0.013 | 0.789±0.016 |  |  |
| Transplant | 13 (0.2) | —— | 1.000 | 0.778±0.139 | 0.648±0.165 |  |  |
| Surgery to LN | | | | | | 228.5 | ＜0.001 |
| Yes | 574 (8.0) | —— | 0.857±0.015 | 0.706±0.020 | 0.644±0.022 |  |  |
| No | 6637 (92.0) | 19.0 (17.9-21.1) | 0.587±0.006 | 0.374±0.006 | 0.291±0.006 |  |  |
| Radiation | | | | | | 60.2 | ＜0.001 |
| Yes | 707 (9.8) | 14.0 (12.7-15.3) | 0.553±0.019 | 0.243±0.018 | 0.142±0.017 |  |  |
| No | 6504 (90.2) | 24.0 (22.4-25.6) | 0.615±0.006 | 0.418±0.006 | 0.337±0.007 |  |  |
| Chemotherapy | | | | | | 46.4 | ＜0.001 |
| Yes | 2613 (36.2) | 18.0 (16.7-19.2) | 0.604±0.010 | 0.319±0.010 | 0.241±0.009 |  |  |
| No | 4598 (63.8) | 27.0 (24.3-29.7) | 0.612±0.007 | 0.450±0.008 | 0.368±0.008 |  |  |
| AFP | | | | | | 197.1 | ＜0.001 |
| Negative | 1949 (27.0) | 41.0 (36.8-45.2) | 0.756±0.010 | 0.533±0.012 | 0.419±0.013 |  |  |
| Positive | 3903 (54.1) | 15.0 (13.8-16.2) | 0.541±0.008 | 0.336±0.008 | 0.271±0.008 |  |  |
| Unknown | 1359 (18.8) | 21.0 (18.1-23.9) | 0.592±0.013 | 0.398±0.014 | 0.317±0.014 |  |  |
| Tumor Size(mm) | | | | | | 1196.0 | ＜0.001 |
| ≤41 | 3060 (42.4) | 58.0 (51.6-64.4) | 0.779±0.008 | 0.591±0.009 | 0.493±0.010 |  |  |
| 42-65 | 1595 (22.1) | 20.0 (17.8-22.2) | 0.626±0.012 | 0.363±0.013 | 0.272±0.013 |  |  |
| ≥66 | 2566 (35.4) | 8.0 (7.3-8.7) | 0.393±0.010 | 0.196±0.008 | 0.140±0.008 |  |  |
| Bone metastasis | | | | | | 278.3 | ＜0.001 |
| Yes | 180 (2.5) | 5.0 (3.5-6.5) | 0.175±0.029 | 0.022±0.012 | 0.000 |  |  |
| No | 7031 (97.5) | 23.0 (21.6-24.4) | 0.620±0.006 | 0.411±0.006 | 0.327±0.006 |  |  |
| Brain metastasis | | | | | | 35.2 | ＜0.001 |
| Yes | 21 (0.3) | 3.0 (0.8-5.2) | 0.143±0.076 | 0.000 | 0.000 |  |  |
| No | 7190 (99.7) | 22.0 (20.6-23.4) | 0.610±0.006 | 0.402±0.006 | 0.321±0.006 |  |  |
| Liver metastasis | | | | | | 133.5 | ＜0.001 |
| Yes | 50 (0.7) | 2.0 (0.9-3.1) | 0.072±0.038 | 0.036±0.032 | 0.000 |  |  |
| No | 7161 (99.3) | 22.0 (20.7-23.3) | 0.613±0.006 | 0.404±0.006 | 0.322±0.006 |  |  |
| Lung metastasis | | | | | | 624.4 | ＜0.001 |
| Yes | 294 (4.1) | 2.0 (1.5-2.5) | 0.133±0.020 | 0.035±0.011 | 0.020±0.009 |  |  |
| No | 6917 (95.9) | 24.0 (22.6-25.4) | 0.629±0.006 | 0.416±0.006 | 0.332±0.006 |  |  |
| Distant LN metastasis | | | | | | 1126.1 | ＜0.001 |
| Yes | 595 (8.3) | 3.0 (2.4-3.6) | 0.152±0.015 | 0.034±0.008 | 0.021±0.006 |  |  |
| No | 6616 (91.7) | 26.0 (24.4-27.6) | 0.650±0.006 | 0.434±0.006 | 0.347±0.007 |  |  |

| \| **Supplementary Table 2** Risk Scores of nomograms corresponding to OS and CSS at 1-,3-, and 5- year. \| \| \| \| \| \| \| --- \| --- \| --- \| --- \| --- \| --- \| \| **1-year OS probability** \| **Total Points** \| **3-year OS probability** \| **Total Points** \| **5-year OS probability** \| **Total Points** \| \| 0.90 \| 56 \| 0.90 \| 10 \| 0.80 \| 32 \| \| 0.80 \| 95 \| 0.80 \| 50 \| 0.70 \| 56 \| \| 0.70 \| 120 \| 0.70 \| 74 \| 0.60 \| 75 \| \| 0.60 \| 139 \| 0.60 \| 93 \| 0.50 \| 91 \| \| 0.50 \| 155 \| 0.50 \| 109 \| 0.40 \| 105 \| \| 0.40 \| 169 \| 0.40 \| 123 \| 0.30 \| 120 \| \| 0.30 \| 183 \| 0.30 \| 137 \| 0.20 \| 135 \| \| 0.20 \| 198 \| 0.20 \| 153 \| 0.10 \| 153 \| \| 0.10 \| 217 \| 0.10 \| 171 \|  \|  \| \| **1-year CSS probability** \| **Total Points** \| **3-year CSS probability** \| **Total Points** \| **5-year CSS probability** \| **Total Points** \| \| 0.90 \| 78 \| 0.90 \| 47 \| 0.90 \| 35 \| \| 0.80 \| 105 \| 0.80 \| 74 \| 0.80 \| 62 \| \| 0.70 \| 122 \| 0.70 \| 91 \| 0.70 \| 79 \| \| 0.60 \| 135 \| 0.60 \| 104 \| 0.60 \| 92 \| \| 0.50 \| 146 \| 0.50 \| 115 \| 0.50 \| 103 \| \| 0.40 \| 156 \| 0.40 \| 125 \| 0.40 \| 113 \| \| 0.30 \| 166 \| 0.30 \| 135 \| 0.30 \| 123 \| \| 0.20 \| 176 \| 0.20 \| 145 \| 0.20 \| 133 \| \| 0.10 \| 189 \| 0.10 \| 158 \| 0.10 \| 146 \|  \| **Supplementary Table 3** The results of nomograms evaluation indicator for OS and CSS. \| \| \| \| \| \| \| \| \| \| \| \| --- \| --- \| --- \| --- \| --- \| --- \| --- \| --- \| --- \| --- \| --- \| \| **Evaluation indicator** \| **C-Index** \| **95%CI** \| ***P* value** \| **AIC** \| ***P* value** \| **BIC** \| ***P* value** \| **C-Index** \| \| \| \| **1-year** \| **3-year** \| **5-year** \| \| OS \|  \| \| \| \| \| \| \| \| \| \| \| Nomogram \|  \| \| \| \| \| \| \| \| \| \| \| Training set \| 0.778 \| (0.777-0.779) \| —— \| 72806.162 \| —— \| 72930.063 \| —— \| 0.788 \| 0.792 \| 0.790 \| \| Validating set \| 0.771 \| (0.770-0.772) \| —— \| 28331.052 \| —— \| 28439.704 \| —— \| 0.792 \| 0.789 \| 0.787 \| \| AJCC staging,7th \|  \| \| \| \| \| \| \| \| \| \| \| Training set \| 0.670 \| (0.652-0.654) \| ＜0.001 \| 75040.765 \| <0.001 \| 75061.415 \| <0.001 \| 0.701 \| 0.701 \| 0.699 \| \| Validating set \| 0.669 \| (0.668-0.670） \| ＜0.001 \| 29195.375 \| <0.001 \| 29213.484 \| <0.001 \| 0.695 \| 0.698 \| 0.696 \| \| SEER staging \|  \| \| \| \| \| \| \| \| \| \| \| Training set \| 0.653 \| (0.652-0.654） \| ＜0.001 \| 75338.806 \| <0.001 \| 75352.573 \| <0.001 \| 0.692 \| 0.694 \| 0.690 \| \| Validating set \| 0.648 \| (0.647-0.649） \| ＜0.001 \| 29383.899 \| <0.001 \| 29395.972 \| <0.001 \| 0.692 \| 0.692 \| 0.687 \| \| CSS \|  \| \| \| \| \| \| \| \| \| \| \| Nomogram \|  \| \| \| \| \| \| \| \| \| \| \| Training set \| 0.792 \| (0.784-0.800) \| —— \| 51444.712 \| —— \| 51541.079 \| —— \| 0.803 \| 0.808 \| 0.806 \| \| Validating set \| 0.787 \| (0.777-0.797) \| —— \| 19820.972 \| —— \| 19881.334 \| —— \| 0.808 \| 0.810 \| 0.809 \| \| AJCC staging,7th \|  \| \| \| \| \| \| \| \| \| \| \| Training set \| 0.693 \| (0.683-0.703) \| ＜0.001 \| 53287.036 \| <0.001 \| 53307.686 \| <0.001 \| 0.722 \| 0.718 \| 0.716 \| \| Validating set \| 0.692 \| (0.678-0.706) \| ＜0.001 \| 20459.249 \| <0.001 \| 20477.357 \| <0.001 \| 0.716 \| 0.718 \| 0.716 \| \| SEER staging \|  \| \| \| \| \| \| \| \| \| \| \| Training set \| 0.670 \| (0.662-0.678) \| ＜0.001 \| 53609.762 \| <0.001 \| 53623.529 \| <0.001 \| 0.696 \| 0.702 \| 0.701 \| \| Validating set \| 0.665 \| (0.651-0.679) \| ＜0.001 \| 20613.495 \| <0.001 \| 20477.357 \| <0.001 \| 0.715 \| 0.713 \| 0.708 \|   **Supplementary Table 4** Nomograms, AJCC stage, and SEER stage were used to predict the 1-, 3-, and 5-year area under the curve (AUC) of OS and CSS in the training and validation set. | | | | | | | |
| --- | --- | --- | --- | --- | --- | --- | --- | --- | --- | --- | --- | --- | --- | --- | --- | --- | --- | --- | --- | --- | --- | --- | --- | --- | --- | --- | --- | --- | --- | --- | --- | --- | --- | --- | --- | --- | --- | --- | --- | --- | --- | --- | --- | --- | --- | --- | --- | --- | --- | --- | --- | --- | --- | --- | --- | --- | --- | --- | --- | --- | --- | --- | --- | --- | --- | --- | --- | --- | --- | --- | --- | --- | --- | --- | --- | --- | --- | --- | --- | --- | --- | --- | --- | --- | --- | --- | --- | --- | --- | --- | --- | --- | --- | --- | --- | --- | --- | --- | --- | --- | --- | --- | --- | --- | --- | --- | --- | --- | --- | --- | --- | --- | --- | --- | --- | --- | --- | --- | --- | --- | --- | --- | --- | --- | --- | --- | --- | --- | --- | --- | --- | --- | --- | --- | --- | --- | --- | --- | --- | --- | --- | --- | --- | --- | --- | --- | --- | --- | --- | --- | --- | --- | --- | --- | --- | --- | --- | --- | --- | --- | --- | --- | --- | --- | --- | --- | --- | --- | --- | --- | --- | --- | --- | --- | --- | --- | --- | --- | --- | --- | --- | --- | --- | --- | --- | --- | --- | --- | --- | --- | --- | --- | --- | --- | --- | --- | --- | --- | --- | --- | --- | --- | --- | --- | --- | --- | --- | --- | --- | --- | --- | --- | --- | --- | --- | --- | --- | --- | --- | --- | --- | --- | --- | --- | --- | --- | --- | --- | --- | --- | --- | --- | --- | --- | --- | --- | --- | --- | --- | --- | --- | --- | --- | --- | --- | --- | --- | --- | --- | --- | --- | --- | --- | --- | --- | --- | --- | --- | --- | --- | --- | --- | --- | --- | --- | --- | --- | --- | --- | --- | --- | --- | --- | --- | --- | --- | --- | --- | --- | --- | --- | --- | --- | --- | --- | --- | --- | --- | --- | --- | --- | --- | --- | --- | --- | --- | --- | --- | --- | --- | --- | --- | --- | --- | --- | --- | --- | --- | --- | --- | --- | --- | --- | --- | --- | --- | --- | --- | --- | --- | --- | --- | --- | --- | --- | --- | --- | --- | --- | --- | --- | --- | --- | --- | --- | --- | --- | --- | --- | --- | --- | --- | --- | --- | --- | --- | --- | --- | --- | --- | --- | --- | --- | --- | --- | --- | --- | --- | --- | --- | --- | --- | --- | --- | --- | --- | --- | --- | --- | --- | --- | --- | --- | --- | --- | --- | --- | --- |
| **Survival** | | **Training set** | | | | | |
|  |  | **1-year** | ***P* value** | **3-year** | ***P* value** | **5-year** | ***P* value** |
| **OS** | **Nomogram** | 0.848 (0.838-0.857) | —— | 0.863 (0.853-0.872) | —— | 0.862 (0.850-0.874) | —— |
|  | **SEER staging** | 0.707 (0.696-0.718) | ＜0.001 | 0.684 (0.673-0.695) | ＜0.001 | 0.671 (0.658-0.684) | ＜0.001 |
|  | **AJCC staging，7th** | 0.729 (0.717-0.741) | ＜0.001 | 0.715 (0.703-0.727) | ＜0.001 | 0.702 (0.687-0.716) | ＜0.001 |
| **CSS** | **Nomogram** | 0.865 (0.856-0.874) | —— | 0.880 (0.871-0.890) | —— | 0.874 (0.862-0.887) | —— |
|  | **SEER staging** | 0.723 (0.710-0.735) | ＜0.001 | 0.697 (0.685-0.709) | ＜0.001 | 0.680 (0.666-0.394) | ＜0.001 |
|  | **AJCC staging，7th** | 0.751 (0.738-0.764) | ＜0.001 | 0.735 (0.722-0.748) | ＜0.001 | 0.717 (0.702-0.732) | ＜0.001 |
|  | | **Validation set** | | | | | |
|  | | **1-year** | ***P* value** | **3-year** | ***P* value** | **5-year** | ***P* value** |
| **OS** | **Nomogram** | 0.845 (0.831-0.860) | —— | 0.850 (0.834-0.865) | —— | 0.861 (0.842-0.879) | —— |
|  | **SEER staging** | 0.706 (0.688-0.723) | ＜0.001 | 0.671 (0.654-0.688) | ＜0.001 | 0.672 (0.653-0.692) | ＜0.001 |
|  | **AJCC staging，7th** | 0.737 (0.719-0.755) | ＜0.001 | 0.709 (0.691-0.727) | ＜0.001 | 0.695 (0.673-0.716) | ＜0.001 |
| **CSS** | **Nomogram** | 0.864 (0.849-0.878) | —— | 0.854 (0.838-0.871) | —— | 0.857 (0.837-0.877) | —— |
|  | **SEER staging** | 0.720 (0.701-0.740) | ＜0.001 | 0.683 (0.664-0.702) | ＜0.001 | 0.684 (0.663-0.706) | ＜0.001 |
|  | **AJCC staging，7th** | 0.759 (0.739-0.778) | ＜0.001 | 0.728 (0.708-0.748) | ＜0.001 | 0.714 (0.691-0.738) | ＜0.001 |

| Supplementary Table 5 Nomograms were used to predict OS and CSS compared with the AJCC stage for 1-, 3-, and 5-year net reclassification improvement (NRI) and integrated discrimination improvement (IDI). | | | | | | |
| --- | --- | --- | --- | --- | --- | --- |
|  | **NRI(AJCC staging,7th)** | | | | | |
|  | **1-year** | ***P* value** | **3-year** | ***P* value** | **5-year** | ***P* value** |
| OS | | | | | | |
| Training set | 0.304 (0.254-0.394) | ＜0.001 | 0.608 (0.533-0.670) | ＜0.001 | 0.658 (0.600-0.739) | ＜0.001 |
| Validating set | 0.340 (0.244-0.434) | ＜0.001 | 0.604 (0.515-0.729) | ＜0.001 | 0.695 (0.602-0.805) | ＜0.001 |
| CSS | | | | | | |
| Training set | 0.268 (0.178-0.307) | ＜0.001 | 0.429 (0.372-0.533) | ＜0.001 | 0.562 (0.498-0.648) | ＜0.001 |
| Validating set | 0.263 (0.176-0.371) | ＜0.001 | 0.502 (0.377-0.593) | ＜0.001 | 0.638 (0.528-0.705) | ＜0.001 |
|  | **IDI(AJCC staging,7th)** | | | | | |
|  | **1-year** |  | **3-year** |  | **5-year** |  |
| OS | | | | | | |
| Training set | 0.145 (0.129-0.162) | ＜0.001 | 0.205 (0.188-0.221) | ＜0.001 | 0.223 (0.200-0.243) | ＜0.001 |
| Validating set | 0.135 (0.116-0.155) | ＜0.001 | 0.182 (0.144-0.207) | ＜0.001 | 0.209 (0.173-0.242) | ＜0.001 |
| CSS | | | | | | |
| Training set | 0.132 (0.116-0.147) | ＜0.001 | 0.207 (0.189-0.226) | ＜0.001 | 0.235 (0.210-0.254) | ＜0.001 |
| Validating set | 0.128 (0.103-0.148) | ＜0.001 | 0.179 (0.146-0.201) | ＜0.001 | 0.214 (0.186-0.247) | ＜0.001 |

| Supplementary Table 6 Nomograms were used to predict OS and CSS compared with the AJCC stage for 1-, 3-, and 5-year net reclassification improvement (NRI) and integrated discrimination improvement (IDI). | | | | | | |
| --- | --- | --- | --- | --- | --- | --- |
|  | **NRI(SEER staging)** | | | | | |
|  | **1-year** | ***P* value** | **3-year** | ***P* value** | **5-year** | ***P* value** |
| OS | | | | | | |
| Training set | 0.356 (0.302-0.448) | ＜0.001 | 0.665 (0.608-0.720) | ＜0.001 | 0.717 (0.664-0.773) | ＜0.001 |
| Validating set | 0.390 (0.275-0.533) | ＜0.001 | 0.661 (0.588-0.778) | ＜0.001 | 0.747 (0.675-0.843) | ＜0.001 |
| CSS | | | | | | |
| Training set | 0.265 (0.187-0.356) | ＜0.001 | 0.561 (0.436-0.622) | ＜0.001 | 0.614 (0.551-0.727) | ＜0.001 |
| Validating set | 0.348 (0.189-0.449) | ＜0.001 | 0.550 (0.448-0.674) | ＜0.001 | 0.679 (0.560-0.777) | ＜0.001 |
|  | **IDI(SEER staging)** | | | | | |
|  | **1-year** |  | **3-year** |  | **5-year** |  |
| OS | | | | | | |
| Training set | 0.175 (0.160-0.192) | ＜0.001 | 0.241 (0.225-0.258) | ＜0.001 | 0.254 (0.235-0.274) | ＜0.001 |
| Validating set | 0.177 (0.151-0.203) | ＜0.001 | 0.229 (0.199-0.262) | ＜0.001 | 0.243 (0.201-0.268) | ＜0.001 |
| CSS | | | | | | |
| Training set | 0.166 (0.152-0.184) | ＜0.001 | 0.255 (0.234-0.273) | ＜0.001 | 0.281 (0.250-0.305) | ＜0.001 |
| Validating set | 0.166 (0.132-0.191) | ＜0.001 | 0.237 (0.208-0.267) | ＜0.001 | 0.261 (0.223-0.294) | ＜0.001 |
